# Supplementary material for: Lokiceratops rangiformis gen. et sp. nov. (Ceratopsidae: Centrosaurinae) from the Campanian Judith River Formation of Montana reveals rapid regional radiations and extreme endemism within centrosaurine dinosaurs
Source: PeerJ. 2024 Jun 20;12:e17224. doi: 10.7717/peerj.17224 (PMC11193970; doi:10.7717/peerj.17224)
Supplement: Supplemental Information 1 [file peerj-12-17224-s001.pdf]

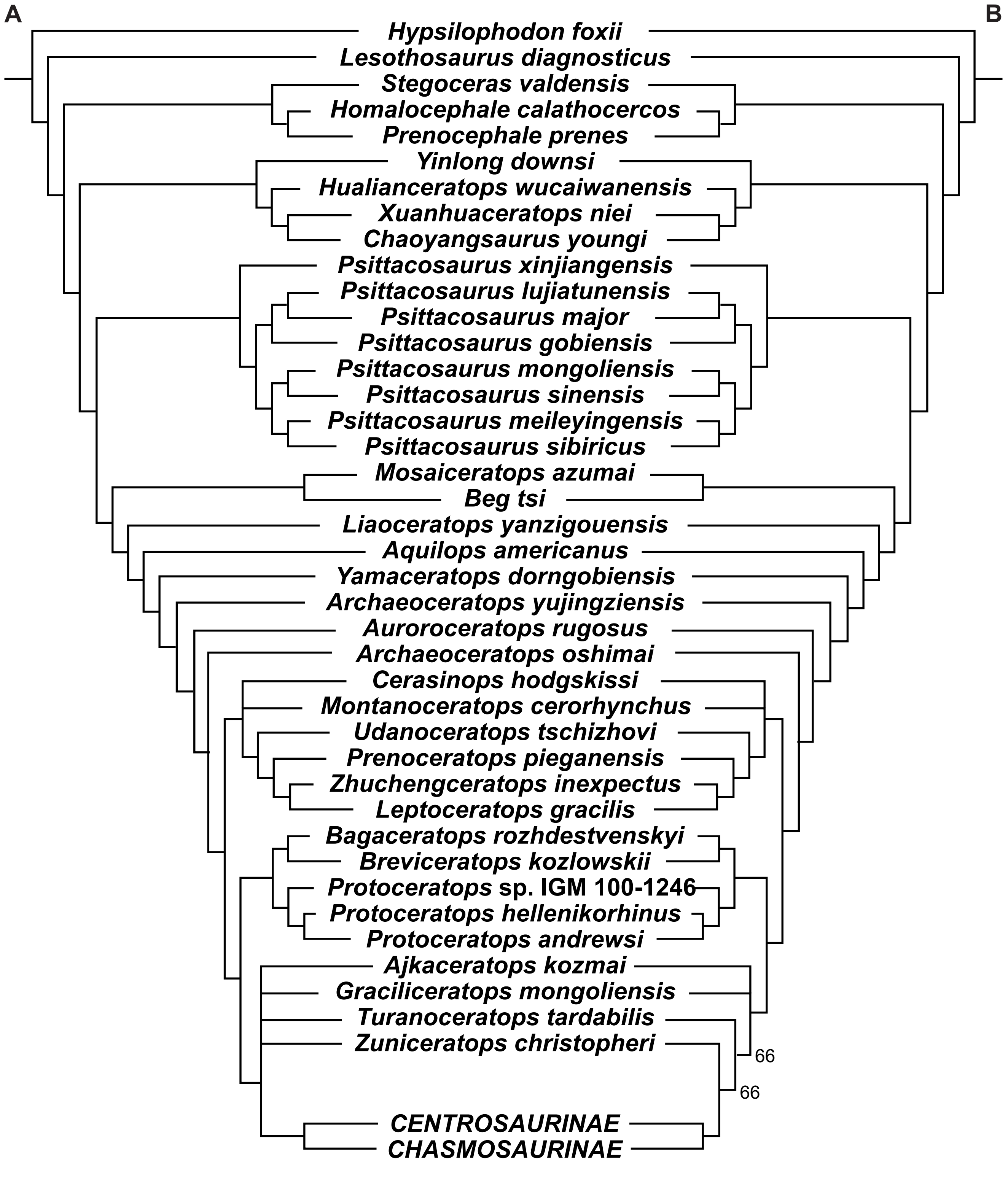

Figure S1. Phylogenetic relationships within Ceratopsia recovered in this study.
(A) Strict consensus tree of 288 MPTs assessing the relationships within Ceratopsia illustrating the phylogenetic position of non-ceratopsid members of the clade (TL: 928; CI: 0.471; RI: 0.898.
(B) 50% Majority-Rules tree with the only nodes scoring less than 100 indicated.

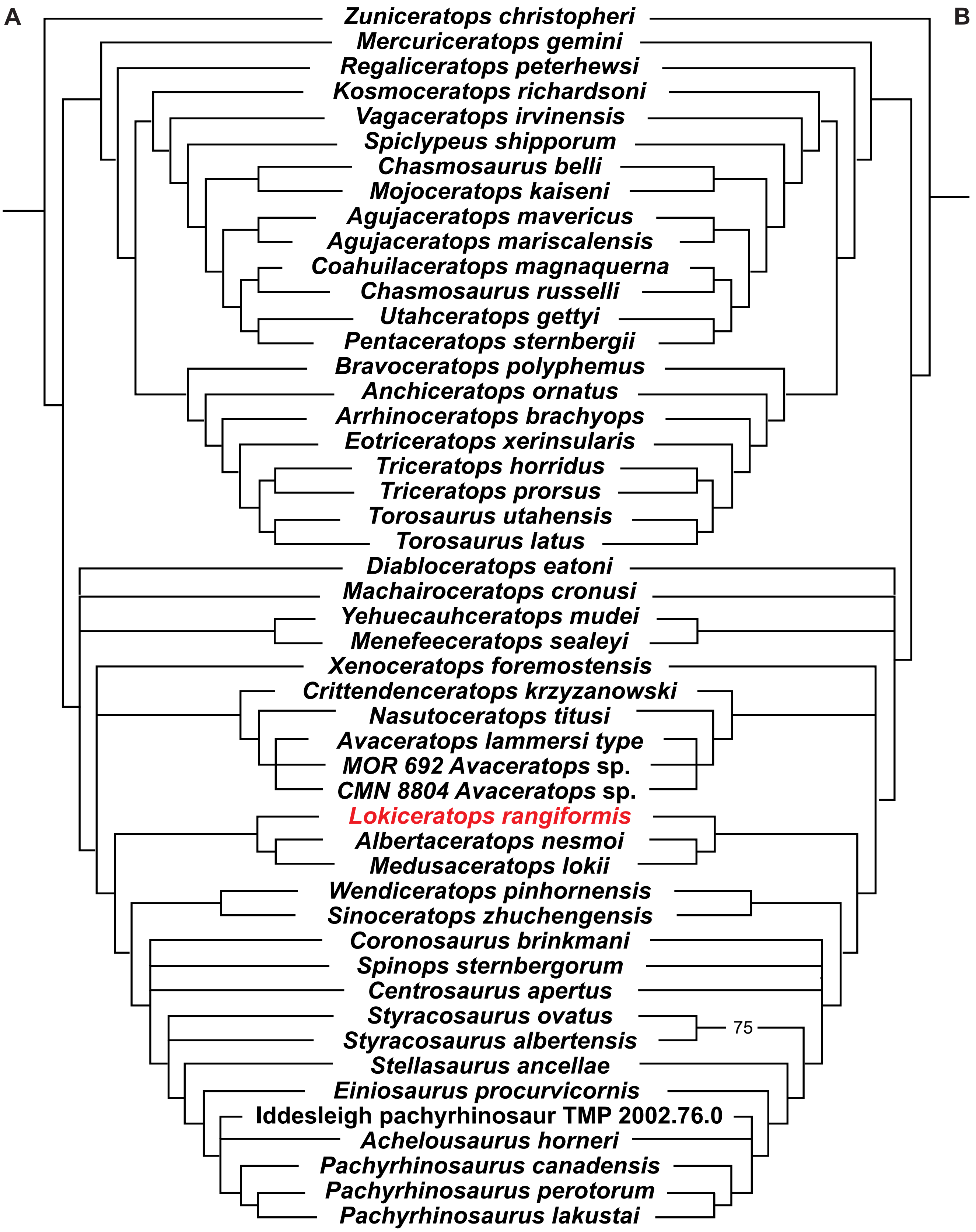

Figure S2. Relationships of *Lokiceratops rangiformis* gen et sp. nov within Ceratopsidae.
(A) Strict consensus tree of 288 MPTs assessing the relationships within Ceratopsidae illustrating the phylogenetic position of *Lokiceratops* (TL: 928; CI: 0.471; RI: 0.898. (B) 50% Majority-Rules tree with the only nodes scoring less than 100 indicated.

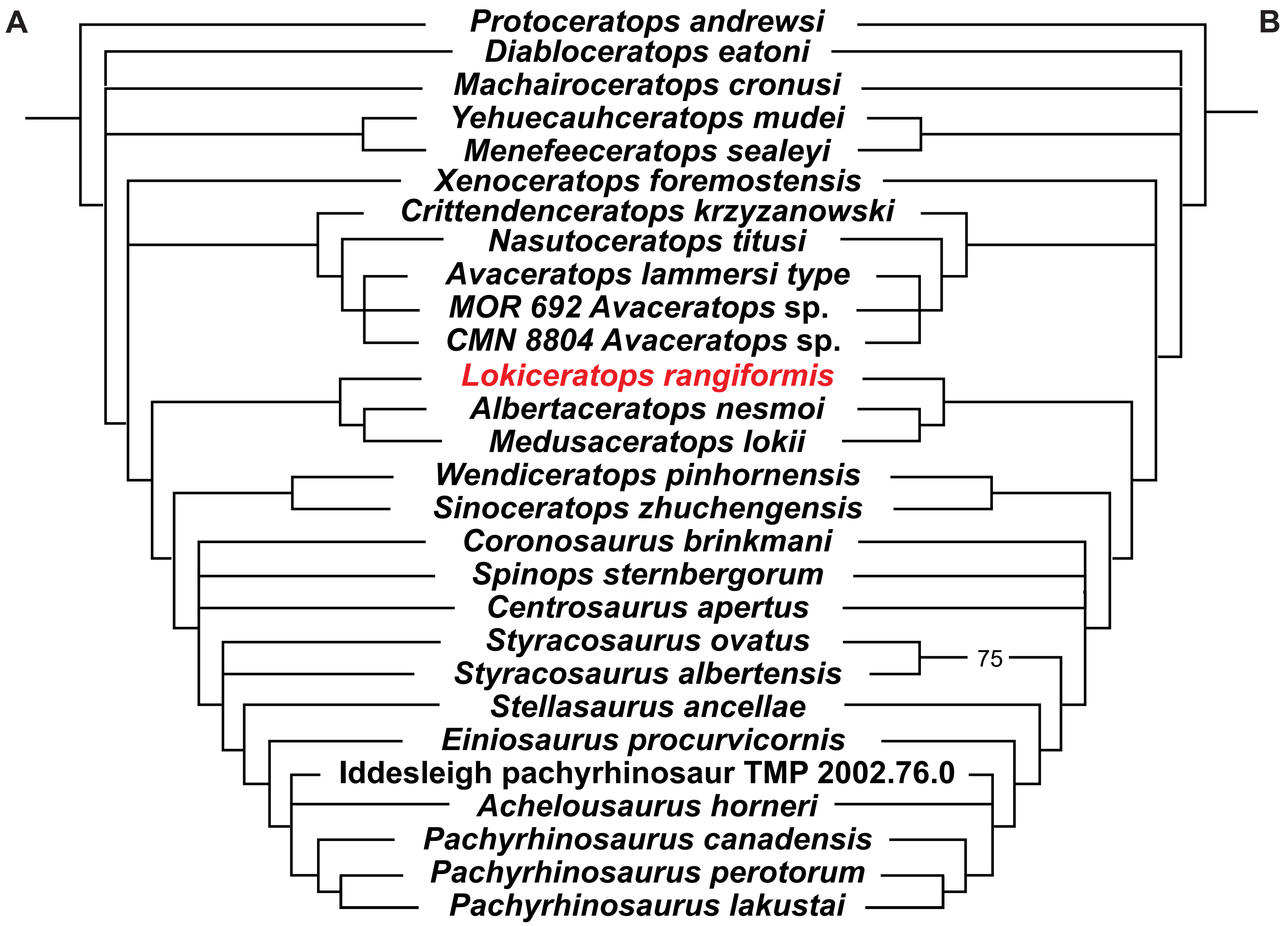

Figure S3. Relationships of *Lokiceratops rangiformis* gen et sp. nov within Centrosaurinae. (A) Strict consensus tree of 64 MPTs assessing the relationships within Centrosaurinae using *Protoceratops andrewsi* as the outgroup illustrating the phylogenetic position *Lokiceratops* (TL: 315; CI: 1.387; RI: 1.026. (B) 50% Majority-Rules tree with the only nodes scoring less than 100 indicated.

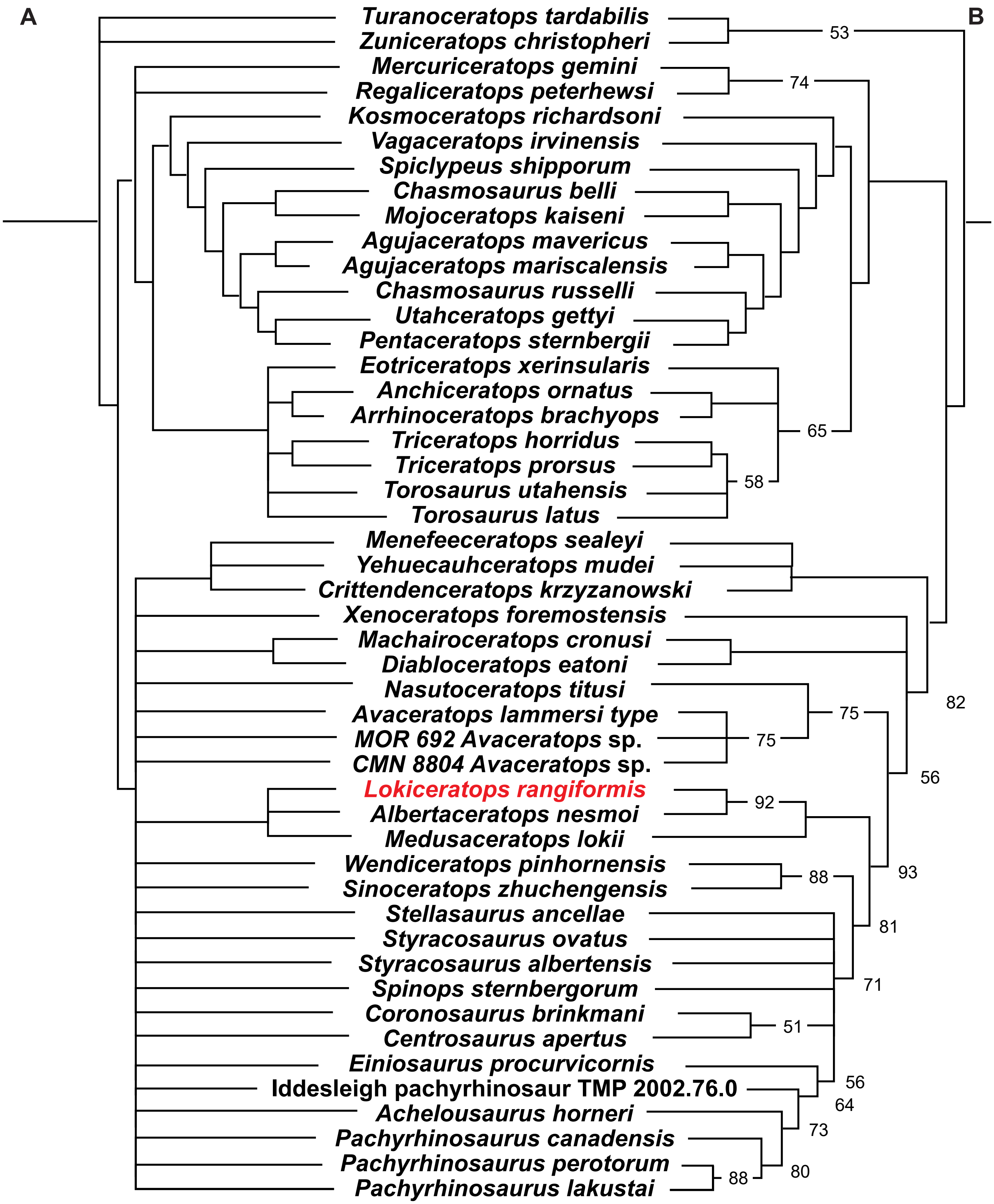

Figure S4. Relationships of *Lokiceratops rangiformis* gen et sp. nov within Ceratopsidae. (A) Strict consensus tree of 10,000 MPTs assessing the relationships within Ceratopsidae without epiossification characters (325-377). (B) 50% Majority-Rules tree with the only nodes scoring less than 100 indicated.

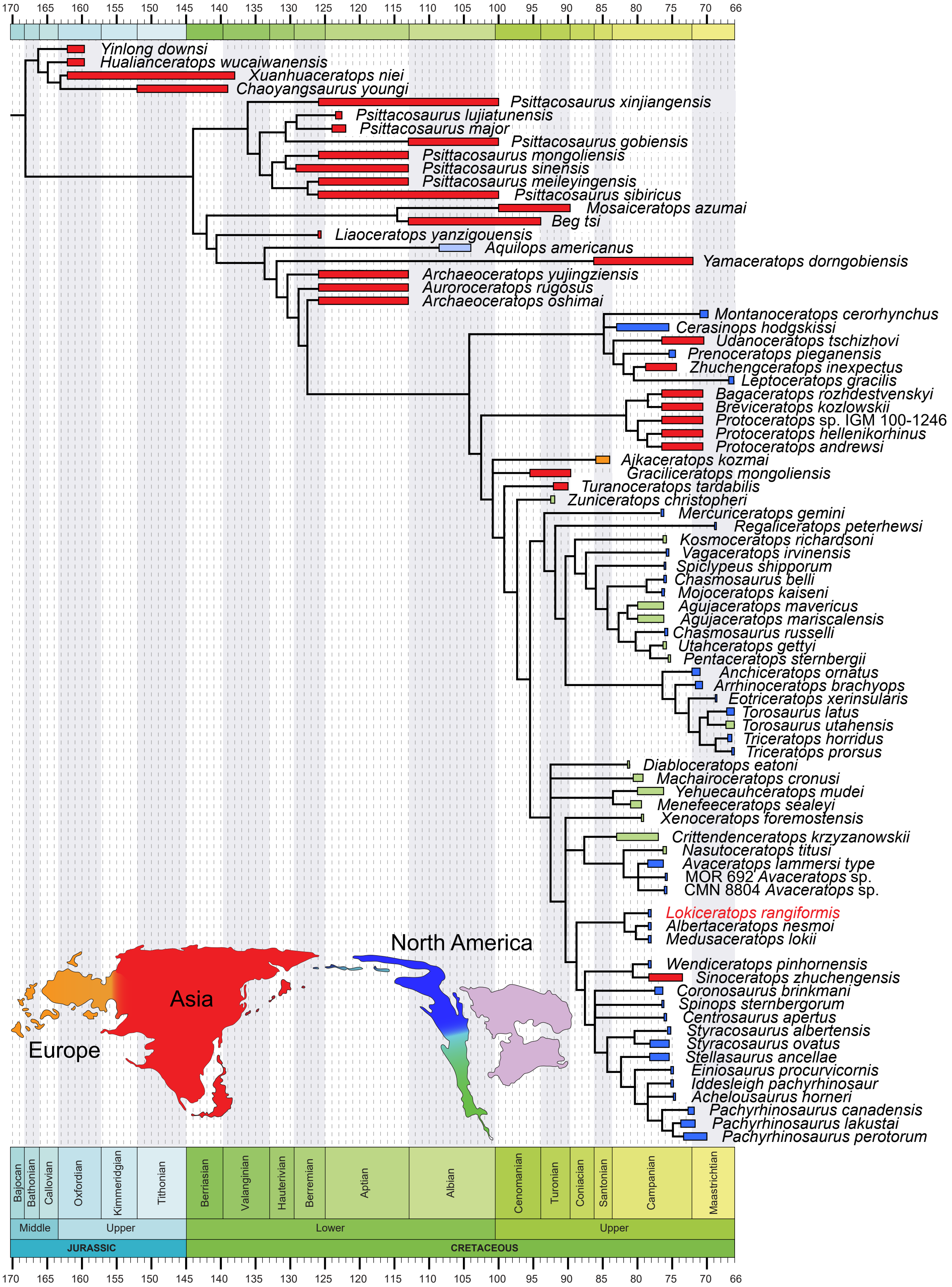

Figure S5. Time-calibrated relationships within Ceratopsia. Relationships within Ceratopsia based on the trees in Fig. S1 and S2 and stratigraphic ranges from Table S1. Possible ranges do not represent absolute temporal position or temporal ranges as many taxa are represented by a single specimen.
