# Supplementary material for: Lokiceratops rangiformis gen. et sp. nov. (Ceratopsidae: Centrosaurinae) from the Campanian Judith River Formation of Montana reveals rapid regional radiations and extreme endemism within centrosaurine dinosaurs
Source: PeerJ. 2024 Jun 20;12:e17224. doi: 10.7717/peerj.17224 (PMC11193970; doi:10.7717/peerj.17224)
Supplement: Supplemental Information 2 [file peerj-12-17224-s002.pdf]

Table S1: Temporal distribution of taxa in the phylogenetic analysis with sources.

Taxon	Formation	Age Range	Sources
Hypsilophodon foxii	Wessex Formation	145–125 Ma	1
Lesothosaurus diagnosticus	upper Elliot Formation	190.8-182.7 Ma	2
Stegoceras valdensis	Dinosaur Park Formation	76.354 ± 0.015-75.639 ± 0.025 Ma	3,4
Homalocephale calathocercos	Nemegt Formation	70.6-68 ma	5,6,7,8
Prenocephale prenes	Nemegt Formation	70.6-68 Ma	5,6,7,8
Yinlong downsi	Shishugou Formation	162.2-159.7 Ma	9
Hualianceratops wucaiwanensis	Shishugou Formation	162.2-159.7 Ma	9
Xuanhuaceratops niei	Houcheng Formation	162.2-138 Ma	10
Chaoyangsaurus youngi	Tuchengzi Formation	152-139 Ma	10
Psittacosaurus xinjiangensis	Tugulu Group	126-100 Ma	11
Psittacosaurus gobiensis	Bayan Gobi Formation	113-100.5 Ma	12
Psittacosaurus major	lowermost Yixian Formation	124-122 Ma	13
Psittacosaurus lujiatunensis	lowermost Yixian Formation	123 Ma	14
Psittacosaurus mongoliensis	Khukhtek Formation	126-113 Ma	13,14
Psittacosaurus sinensis	Doushan Formation	129.4-113 Ma	15,16
Psittacosaurus meileyingensis	Jiufotang Formation	126-113 Ma	17,18
Psittacosaurus sibiricus	Ilek Formation	126-100 Ma	19
Mosaiceratops azumai	Xiaguan Formation	100-89.8 Ma	20
Beg tsi	Ulaanoosh	113-94 Ma	21
Liaoceratops yanzigouensis	Yixian Formation	125.8-125.7 Ma	22
Aquilops americanus	Cloverly Formation	108.5-104 Ma	23
Archaeoceratops yujingziensis	Xinminbao Group	126-113 Ma	24,25
Yamaceratops dorngobiensis	Javhklant Formation	86.3-72.10 Ma	26
Auroroceratops rugosus	Xinminbao Group	126-113 Ma	27
Archaeoceratops oshimai	Xinminbao Group	126-113 Ma	24,25
Cerasinops hodgskissi	Two Medicine Formation	82.419 ± 0.074-75.259 ± 0.027 Ma	28,4
Montanoceratops cerorhynchus	St. Mary River Formation	71.1-69.8 Ma	29
Udanoceratops tschizhovi	Djadochta Formation	76.5-70.5 Ma	5,6,7,8
Prenoceratops pieganensis	Two Medicine Formation	75.259 ± 0.027-74.6 Ma	30,4
Zhuchengceratops inexpectus	Hongtuya Formation	77.3 ± 1.54-73.5 ± 0.3 Ma	31
Leptoceratops gracilis	Scollard Formation	66.9-66.2 Ma	29
Protoceratops hellenikorhinus	Bayan Mandahu Formation	76.5-70.5 Ma	5,6,7,8
Protoceratops andrewsi	Djadochta Formation	76.5-70.5 Ma	5,6,7,8
Protoceratops sp. IGM 100-1246	Djadochta Formation	76.5-70.5 Ma	5,6,7,8
Breviceratops kozlowskii	Barauungoyot Formation	76.5-70.5 Ma	5,6,7,8
Bagaceratops rozhdestvenskyi	Barauungoyot Formation	76.5-70.5 Ma	5,6,7,8
Ajkaceratops kozmai	Csehbánya Formation	86-84 Ma	32
Graciliceratops mongoliensis	Bayan Shireh Formation	95.9-89.6 Ma	33
Turanoceratops tardabilis	Bissekty Formation	92-90 Ma	34
Zuniceratops christopheri	Moreno Hill Formation	92.5-92 Ma	35

Table S1 continued: Temporal distribution of centrosaurine taxa in phylogenetic analysis.

Taxon	Formation	Age Range	Sources
Diabloceratops eatoni	Wahweap Formation	81.42-81.01 Ma (81.27 + 0.36 -0.5)	36
Machairoceratops cronusi	Wahweap Formation	80.68-79.26 Ma (80.06 + 0.62 -0.8)	36
Menefeeceratops sealeyi	Menefee Formation	81-78.5 Ma	37,38,39
Yehuecauhceratops mudei	Aguja Formation	80.0-76.28 ± 0.06 Ma	40,41,42
Xenoceratops foremostensis	Foremost Formation	79.5-78.6 Ma	43,4,44
Crittendenceratops krzyzanowskii	Fort Crittenden Formation	83-77 Ma	47,48.49
Nasutoceratops titusi	Kaiparowits Formation	76.394 ± 0 .040 -75.609 ± 0.015 Ma	50,4
***Avaceratops lammersi* type**	Judith River Formation	78.594 +- 0.024 - 76.24 +- 0.18	51,46,4
***Avaceratops* sp. MOR 692**	Judith River Formation	75.8-75.4 Ma	46,41
***Avaceratops* sp. CMN 8804**	Oldman Formation	76.470 + 0.14/ − 0.084 - 75.8 Ma	52,41,4
Lokiceratops rangiformis	Judith River Formation	78.08 + 0.3 - 0.9 Ma	4
Albertaceratops nesmoi	Oldman Formation	78.28 + 0.2 - 0.9 Ma	4
Medusaceratops lokii	Judith River Formation	78.18 + 0.2 - 0.9 Ma	4
Wendiceratops pinhornensis	Oldman Formation	78.08 + 0.3 - 0.9 Ma	53,4
Sinoceratops zhuchengensis	Hongtuya Formation	77.3 ± 1.54-73.5 ± 0.3 Ma	31
Coronosaurus brinkmani	Oldman Formation	77.5-76.354 ± 0.057 Ma	54,50,55,4
Spinops sternbergorum	Oldman Formation	76.717 ± 0.020-76.354 ± 0.057 Ma	56,4
Centrosaurus apertus	Dinosaur Park Formation	76.470 + 0.14/ − 0.084 - 75.8 Ma	3,4
Styracosaurus albertensis	Dinosaur Park Formation	75.8 -75.3 Ma	3,57,4
Styracosaurus ovatus	Two Medicine Formation	78.2-75.259 ± 0.027 Ma	58,59,4
Stellasaurus ancellae	Two Medicine Formation	78.2-75.259 ± 0.027 Ma	58,59,4
Einiosaurus procurvicornis	Two Medicine Formation	75.259 ± 0.027 Ma	53,51,4
Iddesleigh pachyrhinosaur	Dinosaur Park Formation	75.25-75.22 Ma	61,57,4
Achelousaurus horneri	Two Medicine Formation	75.259 ± 0.027-74.6 Ma	53,51,4
Pachyrhinosaurus lakustai	Wapiti Formation	73.73 ± 0.25-71.89 ± 0.14 Ma	62,29
Pachyrhinosaurus perotorum	Prince Creek Formation	73.4-70.0 ± 0.2 Ma	63,64,55
Pachyrhinosaurus canadensis	Horseshoe Canyon Formation	72.7-71.8 Ma	29
**CPC 279**	Cerro Del Pueblo Fm.	73.63-72.74 Ma	70,71,55

Table S1 concluded: Temporal distribution of chasmosaurine taxa in phylogenetic analysis.

Taxon	Formation	Age Range	Sources
Mercuriceratops gemini	Judith River and Dinosaur Park fms.	76.470 + 0.14/ − 0.084 - 76.354 ± 0.057 Ma	57,4
Regaliceratops peterhewsi	Horseshoe Canyon Formation	68.8-68.5 Ma	65,29
Kosmoceratops richardsoni	Kaiparowits Formation	76.394 ± 0 .040 -75.609 ± 0.015 Ma	50,4
Vagaceratops irvinensis	Dinosaur Park Formation	75.3-75.2 Ma	3,4
Spiclypeus shipporum	Judith River Formation	76.329 ± 0.035-76	66,46,44
Chasmosaurus belli	Dinosaur Park Formation	76.05-75.55 Ma	3,57,4
Mojoceratops kaiseni	Dinosaur Park Formation	76.2-76.1	3,57,4
Agujaceratops mavericus	Aguja Formation	80.0-76.28 ± 0.06 Ma	40,41,42
Agujaceratops mariscalensis	Aguja Formation	80.0-76.28 ± 0.06 Ma	40,41,42
Chasmosaurus russelli	Dinosaur Park Formation	75.1 -74.45	3,4
Utahceratops gettyi	Kaiparowits Formation	76.394 ± 0.040 -75.609 ± 0.015 Ma	50,4
Pentaceratops sternbergii	Fruitland Kirtland Formation	75.5 -75.166 ± 0.014 Ma	67,55,68,4
Anchiceratops ornatus	Horseshoe Canyon Fm.	72.2-70.6 Ma	29
Arrhinoceratops brachyops	Horseshoe Canyon Fm.	71.8-70.5 Ma	29
Eotriceratops xerinsularis	Horseshoe Canyon Fm.	68.9-68.7 Ma	29
Torosaurus latus	Hell Creek Formation	66.9-66.0 Ma	55
Torosaurus utahensis	North Horn Formation	66.9-66.0 Ma	55
Triceratops horridus	Hell Creek Formation	66.5-66.0 Ma	69,55
Triceratops prorsus	Hell Creek Formation	66.9-66.5 Ma	69,55

REFERENCES FOR TABLE S1: Sources of taxon age.

1. **Galton PM. 1974.** The ornithischian dinosaur *Hypsilophodon* from the Wealden of the Isle of Wight. *Bulletin of the British Museum (Natural History) Geology* London. **(5)1**:1-152.
2. **Bordy EM, Abrahams M, Sharman GR, Viglietti PA, Benson RBJ. McPhee BW, Barrett PM, Sciscio L, Condon D, Mundil R, Rademan Z. 2020.** A chronostratigraphic framework for the upper Stormberg Group: implications for the Triassic-Jurassic boundary in southern Africa. *Earth-Science Reviews* **203**:103120.
3. **Ryan MJ, Evans DC. 2005.** Ornithischian Dinosaurs. In: Currie PJ, Koppelhus E, eds. *Dinosaur Provincial Park: A spectacular ancient ecosystem revealed.* Bloomington: Indiana University Press 312-348.
4. **Ramezani J, Beveridge TL, Rogers RR, Eberth DA, Roberts EM. 2022.** Calibrating the zenith of dinosaur diversity in the Campanian of the Western Interior Basin by CA-ID-TIMS U-Pb geochronology. *Scientific Reports* 12(1):16026.
5. **Shuvalov VF. 2000.** The Cretaceous stratigraphy and palaeobiology of Mongolia. In: Benton MJ, Kurochkin EN, Novikov IV, Shishkin MA, Unwin DA, eds. *The Age of Dinosaurs in Russia and Mongolia.* Cambridge University Press, 256–278.
6. **Hicks FJ, Fastovsky D, Nichols DJU, Watabe M. 2001.** Magnetostratigraphic correlation of Late Creta-ceous dinosaur-bearing localities in the Nemegt and Ulan Nuur Basins, Gobi Desert, Mongolia. *Geological Society of America Programs Abstracts* **A**:323.
7. **Dashzeveg D, Dingus L, Loope DB, Swisher III CC, Dulam T, Sweeney MR. 2005.** New Stratigraphic Subdivision, Depositional Environment, and Age Estimate for the Upper Cretaceous Djadokhta Formation, Southern Ulan Nur Basin, Mongolia, *Papers in the Earth and Atmospheric Sciences* 209.

8. **Carpenter K, Hayashi S, Kobayashi Y, Maryańska T, Barsbold R, Sato K, Obata I. 2011.** *Saichania chulsanensis* (Ornithischia, Ankylosauridae) from the upper cretaceous of Mongolia. *Palaeontographica Abteilung A* **294(1-3)**:1-61.
9. **Eberth DA, Xing X, Clark JM. 2010.** Dinosaur death pits from the Jurassic of China. *Palaios* **25(1/2)**:112–125.
10. **Zhao X, Cheng Z, Xu X. 1999.** The Earliest Ceratopsian from the Tuchengzi Formation of Liaoning, China. *Journal of Vertebrate Paleontology* **19(4)**:681–691.
11. **Brinkman DB, Eberth DA, Ryan MJ, Chen P. 2001.** The occurrence of *Psittacosaurus xinjiangensis* Sereno and Chow, 1988 in the Urho area, Junggar Basin, Xinjiang, People's Republic of China. *Canadian Journal of Earth Sciences* **38(12)**:1781-1786.
12. **Sereno PC, Xijin Z, Lin T. 2009.** A new psittacosaur from Inner Mongolia and the parrot-like structure and function of the psittacosaur skull. *Proceedings of the Royal Society B.* **277(1679)**:199–209.
13. **Sereno PC. 2010.** Taxonomy, cranial morphology, and relationships of parrot-beaked dinosaurs (Ceratopsia:Psittacosaurus). In: Ryan MJ, Chinnery-Allgeier BJ, Eberth DA, eds. *New Perspectives on Horned Dinosaurs: The Royal Tyrrell Museum Ceratopsian Symposium.* Bloomington and Indianapolis: Indiana University Press, 21–58.
14. **Hedrick BP, Dodson P. 2013.** Lujiatun Psittacosaurids: Understanding Individual and Taphonomic Variation Using 3D Geometric Morphometrics. *PLoS ONE* **8(8)**:e69265.
15. **Young CC. 1958.** The dinosaur remains of Laiyang, Shantung. *Palaeontologia Sinica Series C* **16**:53–159.
16. **Weishampel DB, Barrett PM, Coria RA, Le Loeuff J, Xu X, Zhao X, Sahni A, Gomani EMP, Noto CR. 2004.** Dinosaur Distribution. In Weishampel DB, Dodson P, Osmólska H, eds. *The Dinosauria, Second Edition.* University of California Press, 517-606.
17. **Eberth DA, Russell DA, Braman DR, Deino AL. 1993.** The age of the dinosaur-bearing sediments at Tebch, Inner Mongolia, People's Republic of China. *Canadian Journal of Earth Sciences*. **30(10)**:2101–2106.
18. **He HY, Wang XL, Zhou ZH, Wang F, Boven A, Shi GH, Zhu RX. 2004.** Timing of the Jiufotang Formation (Jehol Group) in Liaoning, northeastern China, and its implications. *Geophysical Research Letters* **31(13)**:1709.
19. **Leshchinskiy SV, Fayngertz SC, Voronkevich AV, Maschenko AV, Averianov EN, Alexander O. 2000.** Preliminary results of the investigation of the Shestakovo localities of Early Cretaceous vertebrates". In: Komarov AV, ed. *Materials of the Regional Conference of the Geologists of Siberia, Far East and North East of Russia* (in Russian). Tomsk: GalaPress, 363–366.
20. **Zheng W, Jin X, Xu X. 2015.** A psittacosaurid-like basal neoceratopsian from the Upper Cretaceous of central China and its implications for basal ceratopsian evolution. *Scientific reports* **5(14190)**:1-9.
21. **Yu C. Prieto-Marquez A. Chinzorig T. Zorigt B. Norell MA. 2020.** A neoceratopsian dinosaur from the early Cretaceous of Mongolia and the early evolution of ceratopsia. *Communications Biology* **3(10)**:1038.
22. **Zhong Y, Huyskens MH, Yin Q-Z, Wang Y, Ma Q, Xu Y-G. 2021.** High-precision geochronological constraints on the duration of "Dinosaur Pompeii" and the Yixian Formation. *National Science Review* **8(6)**:nwab063.
23. **Burton D, Greenhalgh BW, Britt BB, Kowallis BJ, Elliott Sr WS, et al. 2006** New radiometric ages from the Cedar Mountain Formation, Utah and the Cloverly Formation, Wyoming: implications for contained dinosaur faunas. *Geological Society of America Abstracts with Programs* **38**: 52.
24. **Tang F, Luo Z-X, Zhou Z-H, You H-L, Georgi JA, Tang Z-L, Wang X-Z. 2001.** Biostratigraphy and palaeoenvironment of the dinosaur-bearing sediments in Lower Cretaceous of Mazongshan area, Gansu Province, China. *Cretaceous Research* **22(1)**:115–129.
25. **You H-L, Tanque K, Dodson P. 2010.** A new species of *Archaeoceratops* (Dinosauria: Neoceratopsia) from the Early Cretaceous of the Mazongshan area, northwestern China. In: Ryan MJ, Chinnery-Allgeier BJ, Eberth DA, eds. *New Perspectives on Horned Dinosaurs: The Royal Tyrrell Museum Ceratopsian Symposium.* Bloomington and Indianapolis: Indiana University Press, 59–67.
26. **Eberth DA, Kobayashi Y, Lee Y-N, Mateus O, Therrien F, Zelenitsky DK, Norell MA. 2009.** Assignment of *Yamaceratops dorngobiensis* and associated redbeds at Shine Us Khudag (eastern Gobi, Dorngobi Province, Mongolia) to the redescribed Javkhlant Formation (Upper Cretaceous). *Journal of Vertebrate Paleontology* **29(1)**:295-302.
27. **You H-L, Li D, Ji Q, Lamanna H, Dodson P. 2005.** On a new genus of basal Neoceratopsian dinosaur from the Early Cretaceous of Gansu Province, China. *Acta Geologica Sinica* 79(5):593–597.

28. **Chinnery BJ, Horner JR. 2007.** A new neoceratopsian Dinosaur linking North American and Asian taxa. *Journal of Vertebrate Paleontology* **27**:625-641.
29. **Eberth DA, Kamo SL. 2019.** High-precision U–Pb CA–ID–TIMS dating and chronostratigraphy of the dinosaur-rich Horseshoe Canyon Formation (Upper Cretaceous, Campanian–Maastrichtian), Red Deer River valley, Alberta, Canada. *Canadian Journal of Earth Sciences* **57(10)**:1220-1237.
30. **Chinnery BJ. 2004.** Description of *Prenoceratops pieganensis* gen et sp. nov. (Dinosauria: Neoceratopsia) from the Two Medicine Formation of Montana. *Journal of Vertebrate Paleontology* **24(3)**:572–590.
31. **An W, Kuang H, Liu Y-Q, Peng N, Xu K-M, Xu H, Zhang P, Wang K-B, Chen S-Q, Zhang Y-X. 2016.** Detrital zircon dating and tracing the provenance of dinosaur bone beds from the Late Cretaceous Wangshi Group in Zhucheng, Shandong, East China. *Journal of Palaeogeography* **5(10)**:1016.
32. **Ősi A, Butler RJ. Weishampel DB. 2010.** A Late Cretaceous ceratopsian dinosaur from Europe with Asian affinities. *Nature.* **465(7297)**:466–468.
33. **Kurumada Y, Aoki S, Aoki K, Kato D, Saneyoshi M, Tsogtbaatar K, Windley BF, Ishigaki S. 2020.** Calcite U–Pb age of the Cretaceous vertebrate-bearing Bayn Shire Formation in the Eastern Gobi Desert of Mongolia: usefulness of caliche for age determination. *Terra Nova* **32(4)**:246–252.
34. **Averianov A, Sues H-D. 2012.** Correlation of Late Cretaceous continental vertebrate assemblages in Middle and Central Asia. *Journal of Stratigraphy* **36(2)**:462–485.
35. **Kirkland JI, Smith DK, Wolfe DG. 2005.** Holotype braincase of Nothronychus mckinleyi Kirkland and Wolfe 2001 (Theropoda; Therizinosauridae) from the Upper Cretaceous (Turonian) of west central New Mexico. In Carpenter K ed. *The Carnivorous Dinosaurs Indiana University Pres*s 87-95.
36. **Beveridge TL, Roberts EM, Ramezani J, Titus AL, Eaton JG, Irmis RB, Sertich JJW. 2022.** Refined geochronology and revised stratigraphic nomenclature of the Upper Cretaceous Wahweap Formation, Utah, U.S.A. and the age of early Campanian vertebrates from southern Laramidia, *Palaeogeography, Palaeoclimatology, Palaeoecology* **591**:110876.
37. **Lucas SG, J.A. Spielmann JA, Braman DR, Brister BS, Peters L, McIntosh WC. 2005.** Age of the Cretaceous Menefee Formation, Gallina Hogback, Rio Arriba County, New Mexico. *New Mexico Geological Society, 56th Field Conference Guidebook. Geology of the Chama Basin* **56**: 231–235.
38. **Dalman SG, Lucas SG, Jasinski SE, Lichti AJ, Dodson P. 2021.** The oldest centrosaurine: a new ceratopsid dinosaur (Dinosauria: Ceratopsidae) from the Allison Member of the Menefee Formation (Upper Cretaceous, early Campanian), northwestern New Mexico, USA. *Paläontologische Zeitschrift* **95**:291–335.
39. **McDonald AT, Wolfe DG, Freedman Fowler EA, Gates TA. 2021.** A new brachylophosaurin (Dinosauria: Hadrosauridae) from the Upper Cretaceous Menefee Formation of New Mexico. PeerJ 9:e11084 https://doi.org/10.7717/peerj.11084
40. **Befus KS, Hanson RE, Lehman, TM, Griffen WR. 2008.** Cretaceous basaltic phreatomagmatic volcanism in West Texas: Maar complex at Peña Mountain, Big Bend National Park. Journal of Volcanology and Geothermal Research **173(3–4)**:245-264.
41. **Lehman TM, Wick SL, Barnes KR. 2017.** New specimens of horned dinosaurs from the Aguja Formation of West Texas, and a revision of *Agujaceratops*. *Journal of Systematic Palaeontology* **15(8)**:641-674.
42. **Leslie CE, Peppe DJ, Williamson TE, Heizler M, Jackson M, Atchley SC, Nordt L, Standhardt B. 2018.** Revised age constraints for Late Cretaceous to early Paleocene terrestrial strata from the Dawson Creek section, Big Bend National Park, west Texas. *Geological Society of America Bulletin* **130(7-8)**:1143–1163.
43. **Ryan MJ, Evans DC, Shepherd KM. 2012.** A new ceratopsid from the Foremost Formation (middle Campanian) of Alberta. *Canadian Journal of Earth Science* **49**:1251–1262.
44. **Rogers RR, Eberth DA, Ramezani J. 2023.** The “Judith River–Belly River problem” revisited (Montana-Alberta-Saskatchewan): New perspectives on the correlation of Campanian dinosaur-bearing strata based on a revised stratigraphic model updated with CA-ID-TIMS U-Pb geochronology. Geological Society of America Bulletin doi: https://doi.org/10.1130/B36999.1
45. **Eberth DA. 2005.** The Geology. In: Currie PJ, Koppelhus EB, eds. Dinosaur Provincial Park: A spectacular ancient ecosystem revealed. Bloomington: Indiana University Press, 54–82.
46. **Rogers RR, Kidwell SM, Deino AL, Mitchell JP, Nelson K, Thole JT. 2016.** Age, correlation, and lithostratigraphic revision of the Upper Cretaceous (Campanian) Judith River Formation in its type area (North-Central Montana), with a comparison of low- and high-accommodation alluvial records. Journal of Geology **124(1)**.

47. **Drewes H. 1971.** Mesozoic stratigraphy of the Santa Rita Mountains, southeast Tucson, Arizona: U.S. Geological Survey, Professional Paper 658-C:1-81.
48. **Lucas SG, Heckert AB. 2005.** Distribution, age and correlation of Cretaceous fossil vertebrates from Arizona: New
Mexico Museum of Natural History and Science Bulletin 29, p. 105-110.
49. **Caylor EA, Carrapa B, Sundell K, DeCelles PG, Smith JM. 2021.** Age and deposition of the Fort Crittenden Formation: A window into Late Cretaceous Laramide and Cenozoic tectonics in southeastern Arizona. Bulletin of the Geological Society of America **133(9-10)**:1996-2016.
50. **Roberts EM, Sampson SD, Deino AL, Bowring S. 2013.** The Kaiparowits Formation: a remarkable record of Late Cretaceous terrestrial environments, ecosystems and evolution in Western North America. In: Titus AL, Loewen MA, eds. *At the Top of the Grand Staircase: The Late Cretaceous of Southern Utah*. Bloomington: Indiana University Press, 85–106.
51. **Penkalski P, Dodson P. 1999.** The morphology and systematics of Avaceratops, a primitive horned dinosaur from the Judith River Formation (late Campanian) of North America, with the description of a second skull. *Journal of Vertebrate Paleontology* **19**:692-711.
52. **Ryan MJ, Holmes R, Mallon J, Loewen M, Evans DC. 2017.** A basal ceratopsid (Centrosaurinae: Nasutoceratopsini) from the Oldman Formation (Campanian) of Alberta, Canada. Canadian Journal of Earth Science **54(1)**:1–14.
53. **Evans DC, Ryan MJ. 2015.** Cranial anatomy of *Wendiceratops pinhornensis* gen. et sp. nov., a centrosaurine Ceratopsid (Dinosauria: Ornithischia) from the Oldman Formation (Campanian), Alberta, Canada, and the evolution of Ceratopsid nasal ornamentation. *PLoS ONE* **10(7)**:e0130007.
54. **Ryan MJ, Russell AP. 2005.** A new centrosaurine ceratopsid from the Oldman Formation of Alberta and its implications for centrosaurine taxonomy and systematics. Canadian Journal of Earth Sciences **42(7)**:1369.
55. **Fowler DW. 2017.** Revised geochronology, correlation, and dinosaur stratigraphic ranges of the Santonian-Maastrichtian (Late Cretaceous) formations of the Western Interior of North America. *PLoS ONE* **12(11)**: e0188426.
56. **Farke AA, Ryan MJ, Barrett PM, Tanke DH, Braman DR, Loewen MA, Graham MR. 2011.** A new centrosaurine from the Late Cretaceous of Alberta, Canada, and the evolution of parietal ornamentation in horned dinosaurs. *Acta Palaeontologica Polonica* **56(4)**:691-702.
57. **Mallon JC. 2019.** Competition structured a Late Cretaceous megaherbivorous dinosaur assemblage. Scientific Reports 9:15447. https://doi.org/10.1038/s41598-019-51709-5
58. **Rogers RR, Swisher III CC, Horner JR. 1993.** ^{40}Ar/^{39}Ar age and correlation of the nonmarine Two Medicine Formation (Upper Cretaceous), northwestern Montana, U.S.A. *Canadian Journal of Earth Sciences* **30(5)**:1066-1075.
59. **Wilson JP, Ryan MJ, Evans DC. 2020.** A new, transitional centrosaurine ceratopsid from the Upper Cretaceous Two Medicine Formation of Montana and the evolution of the '*Styracosaurus*-line' dinosaurs. *Royal Society Open Publishing* **7(4)**:200284.
60. **Sampson SD. 1995.** Two new horned dinosaurs from the Upper Cretaceous Two Medicine Formation of Montana; With a phylogenetic analysis of the Centrosaurinae (Ornithischia: Ceratopsidae). *Journal of Vertebrate Paleontology* **15(4)**:743–760.
61. **Ryan MJ, Eberth DA, Brinkman DB, Currie PJ, Tanke DH. 2010.** A New Pachyrhinosaurus-like ceratopsid from the Upper Dinosaur Park Formation (Late Campanian) of Southern Alberta, Canada. In: Ryan MJ, Chinnery-Allgeier B, Eberth DA, eds. New Perspectives on Horned Dinosaurs: The Royal Tyrrell Museum Ceratopsian Symposium. Bloomington: Indiana University Press, 141–155.
62. **Currie PJ, Langston W, Tanke DH. 2008.** A new species of *Pachyrhinosaurus* (Dinosauria, Ceratopsidae) from the Upper Cretaceous of Alberta, Canada. In: Currie PJ, Langston W, Tanke DH, eds. *A New Horned Dinosaur from an Upper Cretaceous Bone Bed in Alberta*. Ottawa, Ontario, Canada: NRC Research Press, 1–108.
63. **Conrad JE, McKee EH, Turrin BD. 1992.** Age of tephra beds at the Ocean Point dinosaur locality, North Slope, Alaska based on K-Ar and ^{40}Ar/^{39}Ar analyses; U.S. Geological Survey Bulletin 1990 Chapter C:1-12.
64. **Fiorillo AR, Tykoski RS. 2012.** A new Maastrichtian species of the centrosaurine ceratopsid *Pachyrhinosaurus* from the North Slope of Alaska: *Acta Palaeontologica Polonica* **57**: 561-573.
65. **Brown CM, Henderson DM. 2015.** A New Horned Dinosaur Reveals Convergent Evolution in Cranial Ornamentation in Ceratopsidae. *Current Biology* **25(12)**:1641–1648.

66. **Mallon JC, Ott CJ, Larson PL, Iuliano EM, Evans DC. 2016.** *Spiclypeus shipporum* gen. et sp. nov., a boldly audacious new chasmosaurine ceratopsid (Dinosauria: Ornithischia) from the Judith River Formation (Upper Cretaceous: Campanian) of Montana, USA. *PLoS ONE* **11(5)**:e0154218.
67. **Fassett JE, Heizler MT. 2017.** An improved new age for the C33n-C32r paleomagnetic reversal, San Juan Basin, NW New Mexico and SW Colorado, In: Karlstrom KE, Gonzales DA, Zimmerer MJ, Heizler M, Ulmer-Scholle DS, eds. *The Geology of the Ouray-Silverton Area*. *New Mexico Geological Society, Guidebook, 68th Field Conference* 115-121. https://doi.org/10.56577/FFC-68.115
68. **Fowler DW, Freedman-Fowler E. 2020.** Transitional evolutionary forms in chasmosaurine ceratopsid dinosaurs: evidence from the Campanian of New Mexico. *PeerJ* 8(e9251).
69. **Scannella JB, Fowler DW, Goodwin MB, Horner JR.** 2014. Evolutionary trends in *Triceratops* from the Hell Creek Formation, Montana. *Proceedings of the National Academy of Sciences* **111**:10245-10250.
70. **Loewen MA, Sampson SD, Lund EK, Farke AA, de Leon C, Aguillón Martínez MC, Rodríguez de la Rosa RA, Getty MA, Eberth DA. 2010.** Horned dinosaurs (Ornithischia: Ceratopsidae) from the Upper Cretaceous (Campanian) Cerro del Pueblo Formation, Coahuila, Mexico. In: Ryan MJ, Chinnery-Allgeier B, Eberth DA, eds. *New Perspectives on Horned Dinosaurs: The Royal Tyrrell Museum Ceratopsian Symposium. Bloomington: Indiana University Press* 99–116.
71. **Eberth DA, Delgado De Jesús CR, Lerbekmo JM, Brinkman DB, Rubén A. Rodríguez-de la Rosa, RA, Sampson SD. 2004.** Cerro del Pueblo Fm (Difunta Group, Upper Cretaceous), Parras Basin, southern Coahuila, Mexico: reference sections, age, and correlation. *Revista Mexicana De Ciencias Geologicas* 21:335-352.
