# Supplementary material for: Lokiceratops rangiformis gen. et sp. nov. (Ceratopsidae: Centrosaurinae) from the Campanian Judith River Formation of Montana reveals rapid regional radiations and extreme endemism within centrosaurine dinosaurs
Source: PeerJ. 2024 Jun 20;12:e17224. doi: 10.7717/peerj.17224 (PMC11193970; doi:10.7717/peerj.17224)
Supplement: Supplemental Information 3 — Characters used in the phylogenetic analysis. [file peerj-12-17224-s003.pdf]

Characters Used in the Phylogenetic Analysis of Ceratopsia

The 425 characters (264 cranial, 61 postcranial, and 100 concerning frill-based ornamentation) used in the phylogenetic analysis are listed below. See article text for specific characters used in the analysis. Recent previous usage of characters is indicated by citations in parentheses. Epiossification scorings are modified from the schemes proposed by Clayton et al. (2009, 2010). For characters 354-425 only activate 23 epiparietal homology characters for either all Ceratopsidae (354-377); Centrosaurinae (378-401); or Chasmosaruinae (402-426). New characters that have not been previously used are indicated in red. Ordered characters are indicated as **ORDERED** and are based on either an observed ontogenetic trajectory within a species of ceratopsian or on inclusion of a character state within another. Ordered characters include 1, 51, 70, 126, 130, 144, 170, 261, 262, 279, 336, and 339.

General Skull

1. **Skull, size relative to the body:**
   (0) – small, shorter than the length of the upper limb bones
   (1) – sub-equal to the length of the upper limb bones
   (2) – large, at least 150% of the length of the upper limb bones
   (Modified from Makovicky and Norell, 2006:1)
   ORDERED
2. **Skull, shape of facial skull in dorsal view:**
   (0) – elongated and ovoid
   (1) – triangular, wide over the jugals
   (Makovicky and Norell, 2006:2)
3. **Skull, relative height of snout (premaxilla) to orbital region:**
   (0) – low
   (1) – deep
   (Modified from Makovicky and Norell, 2006:10)
4. **Skull, naris length compared to basal skull length:**
   (0) – less than 10% of skull length
   (1) – more than 10% of skull length
   (Modified from Makovicky and Norell, 2006:19)
5. **Skull, preorbital skull length compared to overall skull length (disregarding epiossifications):**
   (0) – more than 41%
   (1) – 35 to 40%
   (2) – 30 to 34%
   (3) – less than 29%
   (Modified from Makovicky and Norell, 2006:4; Sereno 2010:1)
6. **Palatomaxillary foramen:**
   (0) – absent
   (1) – present
   (Hatcher et al., 1907)
7. **Position of anterior end of choana on palate:**
   (0) – anterior to maxillary tooth row
   (1) – or even with maxillary tooth row
   (Makovicky & Norell, 2006:20)
8. **Skull, suborbital fenestra or pterygopalatine foramen size:**
   (0) – large
   (1) – diminutive
   (Modified from Makovicky and Norell, 2006:45

Rostral

9. **Rostral, presence of the rostral forming beak:**
   (0) – absent
   (1) – present
   (Modified from Makovicky and Norell, 2006:6)

10. Rostral, shape of body and extent of dorsal and ventral processes:
(0) – triangular in lateral view, lacks discrete dorsal and ventral processes
(1) – elongated ventral process short, broad dorsal and ventral processes
(2) – elongated dorsal and ventral processes extending from the rostral body with prominent concavity on the posterior margin invaginated at least 33% or anteroposterior length of the element
(Modified from Dodson et al., 2004:1; Sampson et al., 2010:1; Farke et al., 2011:1)

11. Rostral, presence of ventral (buccal) process:
(0) – absent
(1) – present
(Modified from Makovicky and Norell, 2006:7)

12. Rostral, contact with nasal:
(0) – present
(1) – absent
(Sereno 2010:4)

13. Rostral, shape in occlusal view:
(0) – broad and rounded anteriorly, "U" shaped
(1) – acute, "V"-shaped
(New Character)

14. Rostral, shape of ventral margin in lateral view:
(0) – straight or flat
(1) – ventrally concave
(New Character)

Premaxilla

15. Premaxilla, position of ventral border of external naris (endonaris) compared to laterotemporal fenestra:
(0) – significantly above ventral rim of laterotemporal fenestra
(1) – about the level of ventral rim of laterotemporal fenestra
(2) – significantly below ventral rim of laterotemporal fenestra
(Modified from Makovicky & Norell, 2006:16)
ORDERED

16. Premaxilla, position of ventral border of nares compared to orbit:
(0) – is positioned well above the ventral extent of the orbit
(1) – extends to just above the ventral extent of the orbit but not below
(2) – extends below the orbit
(Modified from Makovicky & Norell, 2006:15)

17. Premaxilla, position of ventral border of nares compared to maxillary alveolar tooth margin:
(0) – extends below the orbit but not to the alveolar margin
(1) – extends to the alveolar margin
(Modified from Makovicky & Norell, 2006:15)

18. Premaxilla, presence of external narial fossa:
(0) – absent
(1) – present as slight outline on lateral surface of premaxilla
(2) – present as a discrete invagination
(Modified from Makovicky and Norell, 2006:17; Sampson et al., 2010:4)

19. Premaxilla, external narial fossa, shape:
(0) – tall, hemicircular
(1) – anteriorly elongate
(Modified from Sampson et al., 2010:4; Farke et al., 2011:2)

20. Premaxilla, osteonarial complex, size:
(0) – small, occupies 50% or less height of snout
(1) – large, occupies entire height of snout
(Modified after Gregory & Mook, 1925; Brown & Schlaikjer, 1940a)

21. **Premaxilla, presence of premaxillary septum:**
(0) – absent
(1) – present
(Forster, 1990)
22. **Premaxillary septum, nasal contribution:**
(0) – septum formed by premaxilla only
(1) – septum formed by premaxilla and nasal
(Sampson et al., 2010:5; Farke et al., 2011:3)
23. **Premaxilla, narial strut:**
(0) – absent
(1) – present
(Farke et al., 2011:4)
24. **Premaxilla, narial strut:**
(0) – present, nascent
(1) – present, well-developed
(Modified after Farke et al., 2011:4)
25. **Premaxilla, narial strut orientation:**
(0) – anteriorly inclined
(1) – posteriorly inclined
(Dodson et al., 2004:6; Sampson et al., 2010:7)
26. **Premaxilla, septal flange:**
(0) – absent
(1) – fin of bone posterior to narial strut present
(Modified from Forster et al., 1993:1)
27. **Premaxilla, septal flange length:**
(0) – spans entire posterior margin of narial strut
(1) – restricted to ventral portion of narial strut
(Modified from Forster et al., 1993: 1)
28. **Premaxilla, septal fossa:**
(0) – absent
(1) – present
(Farke et al., 2011:5)
29. **Premaxilla, septal recess:**
(0) – absent
(1) – present
(Modified from Dodson et al., 2004:9; Farke et al., 2011:7)
30. **Premaxilla, accessory strut in septal fossa and septal recess:**
(0) – absent
(1) – present
(Sampson et al., 2010:12)
31. **Premaxilla, endonarial process:**
(0) – absent
(1) – (triangular process) present
(Forster, 1990:21; Sampson et al., 2010:13; Farke et al., 2011:6)
32. **Premaxilla, endonarial process shape in lateral view:**
(0) – square with straight margins
(1) – triangular with concave margins
(Sampson et al., 2010:14)
33. **Premaxilla, endonarial process recess:**
(0) – absent
(1) – present
(Forster, 1990; Sampson et al., 2010:15)
34. **Premaxilla, ectonarial recess**
(0) – absent
(1) – present
(Sampson et al., 2010:16)

35. Premaxilla, endonarial platform:
(0) – present
(1) – absent
(New Character)

36. Premaxilla, ventral palatal region shape:
(0) – flat in ventral view
(1) – vaulted dorsally
(Modified from Makovicky and Norell, 2006:9)

37. Premaxilla, ventral extent compared to level of alveolar margin:
(0) – above or at the level of alveolar margin of maxilla
(1) – expanded ventrally to extend well below alveolar margin of maxilla
(Modified after Makovicky & Norell, 2006:12; Farke et al., 2011:9)

38. Premaxilla, presence of ventral angle:
(0) – absent
(1) – present
(Forster, 1990; Sampson et al., 2010:17; Farke et al., 2011:8)

39. Premaxilla, composition of expanded ventral angle:
(0) – premaxilla and maxilla
(1) – premaxilla
(Modified after Makovicky & Norell, 2006:12; Farke et al., 2011:10)

40. Premaxilla, form of distal end of posterioventral process:
(0) – broad and uniform in width
(1) – broad and expanded, paddle shaped
(2) – thin
(Modified from Forster et al., 1993:7; Farke et al., 2011:12)

41. Premaxilla, form of thin distal end of posterioventral process:
(0) – lancelate or squared
(1) – forked
(Modified from Forster et al., 1993:7; Sampson et al., 2010:18; Farke et al., 2011; 11)

42. Premaxilla, presence of an ornamental spike on the anterodorsal surface between the rostral and nasal:
(0) – absent
(1) – present, distinct bump
(2) – present, pointed hook
(New Character)
ORDERED

43. Premaxilla, nasal dorsal contact in dorsal view:
(0) – premaxillae insert between nasals
(1) – nasals insert between premaxillae
(Sampson et al., 2010:20, Farke et al, 2011:13)

44. Premaxilla-nasal dorsal contact, length in dorsal view:
(0) – short
(1) – premaxilla and nasal contact for at least 30% of the length of the nasal
(New Character)

45. Premaxilla, premaxilla-nasal contact in dorsal view:
(0) – premaxilla tapers posteriorly
(1) – premaxilla broadens posteriorly
(New Character)

46. Premaxilla, contribution of premaxilla to posterior margin of external naris
(0) – less than 50%
(1) – more than 55%
(New Character)

47. Premaxilla, premaxilla-prefrontal contact:
(0) – absent
(1) – present
(Modified from Makovicky and Norell, 2006:11)

48. Premaxilla, premaxilla-jugal contact or approximation:
(0) – absent
(1) – present
(Sereno 2010:7)

Accessory Antorbital Fenestra

49. Accessory antorbital fenestra, presence between naris and antorbital fenestra:
(0) – absent
(1) – present
(Forster 1990; 15; Kirkland and Deblieux, 2009; Sampson et al., 2010:21; Farke et al., 2011:14; Farke et al., 2014:143)

50. Accessory antorbital fenestra size.
(0) – pronounced, penetration of nasal cavity visible in lateral view
(1) – slight penetration, nasal cavity not visible in lateral view
(Sampson et al., 2010:22; Farke et al., 2011:15)

51. Accessory antorbital fenestra, nasal contribution to dorsal margin:
(0) – nasal is excluded from accessory antorbital fenestra
(1) – nasal makes up less than 50%
(2) – nasal makes up more than 60%
(New Character)
ORDERED

Antorbital Fenestra

52. Antorbital fenestra:
(0) – present
(1) – nascent or vestigial
(2) – absent
(New Character)

53. Antorbital fenestra exposure in lateral view:
(0) – well exposed in lateral view
(1) – small slit like opening between premaxilla and maxilla
(New Character)

54. External antorbital fenestra/fossa, size:
(0) – large, 15% or more length of body of maxilla
(1) – greatly reduced, less than 10% length of body of maxilla
(Modified from Granger & Gregory, 1923; Chinnery & Weishampel, 1998; Sampson et al., 2010:23; Farke et al., 2011:16; Makovicky and Norell, 2006:23)

Maxilla

55. Maxilla, shape of oral margin in ventral view:
(0) – sinuous, laterally concave
(1) – relatively straight, oral margins converge anteriorly
(Modified Makovicky & Norell, 2006:13)

56. Maxilla, ventral displacement of tooth row relative to ventral surface of premaxilla:
(0) – absent
(1) – present
(Sampson et al., 2010:24; Farke et al., 2011:17)

57. Maxilla, tooth row diastema:
(0) – absent
(1) – present
(Sampson et al., 2010:25)

58. Maxilla, maxillary cavity opening on the medial surface of the ascending process and penetrating into the interior of the bone:
(0) – absent
(1) – present
(Sampson et al., 2010:26; Farke et al., 2011:18)

59. **Maxilla, internal maxillary fossa on medial surface dorsal to the palatal process:**
(0) – absent
(1) – present
(New Character)

60. **Maxilla, length of posterior (alveolar) ramus compared to toothrow length:**
(0) – short, 25% or less
(1) – long, 30% or more
(Sereno, 1999)

61. **Maxilla, ectotopterygoid/pterygoid complex exposure on posterior ramus:**
(0) – covers entire dorsal surface and laps onto lateral surface
(1) – reduced to the posterior-most portion
(Farke et al., 2011:19)

62. **Maxilla, relation to vomers:**
(0) – separated by vomers
(1) – contact each other in palatal view; tip of vomer obscured from view
(Modified from Makovicky and Norell, 2006:22)

63. **Maxilla, eminence or tubercle on the rim of the buccal emargination of the maxilla near the junction with the jugal:**
(0) – absent
(1) – present
(Modified from Makovicky and Norell, 2006:24)

64. **Maxilla, presence of maxillary fossa:**
(0) – absent
(1) – present
(Sereno 2010:8)

Nasal

65. **Nasal, anterior end relative to external naris:**
(0) – dorsal to or posterior to
(1) – anterior to
(Modified from Makovicky and Norell, 2006:14)

66. **Nasal, dorsal surface nasal-nasal contact transverse section:**
(0) – concave
(1) – flat
(2) – peaked or convex
(Modified from Farke et al., 2014:137)

67. **Nasal, ornamentation in adult:**
(0) – absent
(1) – present
(Forster, 1990; Sampson et al., 2010:27)

68. **Nasal, presence of pronounced ornamentation type in adult:**
(0) – absent or low prominence
(1) – distinct horncore
(2) – pachyostotic boss
(Modified from Forster, 1990: 26,27,28,126; Sampson et al., 2010:28; Farke et al., 2011:20)

69. **Nasal, pachyostotic boss development:**
(0) – nasal boss clearly distinct from postorbital bosses
(1) – coalesced into a continuous boss with little differentiation from postorbital bosses
(New Character)

70. Nasal, posterior extent of nasal ornamentation:
(0) – posterior end of nasal ornamentation is well anterior to the posterior end of the antorbital fenestra
(1) – extends to about the posterior end of the antorbital fenestra
(2) – extends well posterior to antorbital fenestra but still nearly an orbit length separates posterior extent and antorbital buttress
(3) – extends to just anterior to antorbital buttress
(4) – extends posterior to antorbital buttress
(Modified from Evans et al., 2015)
ORDERED

71. Nasal, ornamentation position, measured perpendicular to the toothrow:
(0) – centered posterior to anterior end of toothrow
(1) – centered anterior to anterior end of toothrow
(Modified after Forster, 1990; Sampson et al., 2010:29)

72. Nasal ornamentation base, cross sectional shape:
(0) – sub-circular to ovoid
(1) – mediolaterally compressed, width 66% anteroposterior length of base
(2) – elongate width less than 33% anteroposterior length of base
(3) – covers entire nasal
(New Character)

73. Nasal ornamentation, height compared to the orbit:
(0) – present but less than or equal to the height of the orbit
(1) – short, 1 to 2 times taller than the orbit
(2) – elongated, more than 3 times taller than the orbit
(New Character)

74. Nasal ornamentation, curvature of horncore:
(0) – no curvature
(1) – curved
(New Character)

75. Nasal ornamentation, anterior projection of premaxillary process of nasal forms spike (rostral comb):
(0) – absent
(1) – present
(New Character)

76. Nasal, epinasal ossification:
(0) – absent
(1) – present
(Lehman, 1990; Forster, 1990; Farke et al., 2011:21)

77. Nasal, dorsoventral thickness at apex of external naris:
(0) – thin, uniform thickness from medial to lateral
(1) – thick, ventral surface of nasal relatively flat
(New Character)

78. Nasal, vaulting of internal surface in lateral view:
(0) – absent, flat
(1) – present
(New Character)

79. Nasal, narial spine:
(0) – absent
(1) – present as a distinct inflection in the posterior or posteriomedial surface of the naris
(Modified from Lehman, 1990; Forster, 1990:22; Sampson et al., 2010:30, Farke et al., 2011:22)

80. Nasal, contribution to narial spine:
(0) – forms less than 60 % of narial spine
(1) – forms greater than 70% of narial spine
(New Character)

81. Nasal, narial spine orientation:
(0) – nascent process medially directed
(1) – process anteriorly directed
(New Character)

Orbit

82. Orbital region, facial skeleton, dorsoventral depth:
(0) – deep, maxilla dorsally expanded, posterior ramus of maxilla entirely visible
(1) – shallow, maxilla dorsoventrally low, posterior ramus of maxilla partially covered by jugal
(Sampson et al., 2010:31)

83. Orbit, orientation:
(0) – directed anterolaterally
(1) – directed laterally
(Sampson et al., 2010:32)

84. Orbit diameter, relative to basal skull length:
(0) – more than 18%
(1) – less than 15%
(Makovicky & Norell, 2006:3; Sampson et al., 2010:33)

85. Orbit, contribution of lacrimal to anterior orbital margin:
(0) – forms 50% or more of the margin
(1) – forms 40% or less of the margin
(Brown and Schlaikjer, 1940a; Sampson et al., 2010:34)

86. Orbit, lacrimal canal fenestra
(0) – absent
(1) – present
(Sereno 2010:10)

Postorbital

87. Postorbital, contribution to supratemporal fenestra:
(0) – absent
(1) – present
(Makovicky & Norell, 2006:33)

88. Postorbital, orbital ornamentation in adults:
(0) – absent
(1) – low ornamentation
(2) – horncore
(Modified from Forster et al., 1993:5; Makovicky and Norell, 2006:31; Sampson et al., 2010:3; Farke et al., 2011:25 and 28)

89. Postorbital, form of low orbital ornamentation in adults:
(0) – reduced to low rugosity
(1) – pachyostotic boss
(Modified from Forster et al., 1993:5; Sampson et al., 2010:3; Farke et al., 2011:25 and 28)

90. Postorbital, type of subadult orbital ornamentation:
(0) – pointed apex, horncore at least as tall as long anteroposteriorly
(1) – rounded apex, horncore longer anteroposteriorly than high
(Modified from Sampson, 1995a:10; Farke et al., 2011:24)

91. Postorbital ornamentation, size relative to the nasal ornamentation:
(0) – larger than nasal ornamentation
(1) – smaller than nasal ornamentation
(New Character)

92. Postorbital, position of orbital ornamentation:
(0) – centered anterodorsal or dorsal to orbit, narrow base with posterior margin of orbital horn extending to or only slightly behind posterior margin of orbit
(1) – centered posteriodorsal to orbit, broad base with posterior margin of orbital horn extending well behind posterior orbit
(Modified from Lehman, 1996; Sampson et al., 2010:37; Farke et al., 2011:26)

93. Postorbital, orientation of orbital horncore base:
(0) – dorsally directed
(1) – dorsolaterally directed
(Sampson et al., 2010:38; Farke et al., 2011:27)

94. Postorbital, curvature of orbital horncore of adults in lateral view:
(0) – posteriorly curved
(1) – anteriorly curved
(2) – straight
(Modified from Forster et al., 1993:2; Sampson et al., 2010:40; Farke et al., 2011:29)

95. Postorbital, curvature of orbital horncore of adults in anterior view:
(0) – medially curved
(1) – laterally curved
(2) – straight
(Sampson et al., 2010:41, Farke et al., 2011:30)

96. Cornual sinuses near the base of orbital ornamentation:
(0) – absent
(1) – present
(Farke, 2006)

97. Extent of cornual sinuses in base of orbital ornamentation:
(0) – sinus space invades frontal and parietal only
(1) – sinus space enters postorbital
(Modified from Forster 1990: 123; Farke, 2006; Sampson et al., 2010:36; Farke et al., 2011:23)

98. Postorbital, contribution to laterotemporal fenestra:
(0) – postorbital participation in margin
(1) – postorbital excluded from margin
(Makovicky & Norell, 2006:34)

99. Postorbital, separation from laterotemporal fenestra:
(0) – postorbital excluded from margin slightly by narrow strip of jugal
(1) – jugal–squamosal contact very wide and postorbital situated far from fenestra
(Makovicky & Norell, 2006:34; Sampson et al., 2010:42)

Palpebal

100. Palpebral, shape and attachment:
(0) – rod-like, articulates with prefrontal only at its base, ligamentous attachment
(1) – blocky, fully sutured to and fused into prefrontal and frontal around dorsal orbital margin
(Granger & Gregory, 1923; modified from Forster 1990:31; Makovicky and Norell, 2006:25; Sampson et al., 2010:43; Farke et al., 2011:32)

101. Palpebral, antorbital buttress:
(0) – absent
(1) – present
(Ostrom and Wellnhofer, 1986; Sampson, 1995:7: Sampson et al., 2010:44; Farke et al., 2011:33)

102. Palpebral, extent of antorbital buttress:
(0) – restricted to anterodorsal orbital margin
(1) – present along entire anterior orbit al margin
(Ostrom and Wellnhofer, 1986; Sampson, 1995a: Currie et al., 2008; Sampson et al., 2010:45; Farke et al., 2011:34)

Jugal

103. Jugal, shape of jugal flange:
(0) – free jugal flange absent
(1) – broad based, narrows distally (Delta-shaped)
(2) – narrowed base, anterior and posterior margins subparallel (narrow-waisted)
(New Character)

104. Jugal, thickening of jugal flange along its anterior edge:
(0) – present
(1) – absent, plate-like flange
(New Character)

105. Jugal, size and orientation of jugal ventral process:
(0) – projects strongly posteriorly, distal end does not extend below maxillary tooth row
(1) – projects more ventrally, distal end extends below maxillary tooth row
(Makovicky 2001:22; Sampson et al., 2010:46; Farke et al., 2011:35)

106. Jugal, suborbital ramus depth:
(0) – not as deep as subtemporal ramus
(1) – as deep or deeper than subtemporal ramus
(Modified from Makovicky and Norell, 2006:28)

107. Jugal infratemporal process:
(0) – absent
(1) – present
(Modified from Brown and Schlaikjer, 1940a; modified from Forster, 1990:62; Sampson et al., 2010:47; Farke et al., 2011:36)

108. Jugal infratemporal process:
(0) – long
(1) – short
(Modified from Brown and Schlaikjer, 1940a; modified from Forster, 1990:62; Sampson et al., 2010:47; Farke et al., 2011:36)

109. Jugal infratemporal process shape in dorsal view:
(0) – curved
(1) – straight
(New Character)

110. Jugal surface texture of infratemporal process and infratemporal bar:
(0) – smooth
(1) – textured
(New Character)

111. Jugal type of texture on infratemporal process and infratemporal bar:
(0) – grooved
(1) – nodular
(New Character)

112. Jugal–lacrimal contact:
(0) – very limited
(1) – extensive
(Makovicky & Norell, 2006:26; Sampson et al., 2010:48)

113. Jugal horn orientation:
(0) – jugal ornamentation lacking
(1) – laterally directed
(2) – ventrolaterally directed
(Makovicky & Norell, 2006:27)
ORDERED

Epijugal

114. Epijugal:
(0) – absent
(1) – present
(Makovicky and Norell, 2006:29)

115. Epijugal, position of epijugal on jugal:
(0) – covers most of the posterodistal jugal
(1) – restricted to just the distal most tip of the jugal
(Modified from Makovicky and Norell, 2006:30)

116. Epijugal cross-sectional shape:
(0) – large blade like triangle with obtuse angle oriented towards quadratojugal
(1) – roughly equilateral in shape
(Sampson et al., 2010:49; Farke et al., 2011:37)

117. Epijugal length of horn:
(0) – as long or longer than wide
(1) – substantially shorter than wide
(Modified from Sampson et al., 2010:50)

118. Epijugal, extent of horn length:
(0) – as long as wide
(1) – at least 1.5 times longer than wide
(New Character)

Quadratojugal

119. Quadratojugal shape:
(0) – mediolaterally compressed
(1) – transversely expanded medial to jugal
(2) – a squat mediolaterally flattened pedestal
(Modified from Makovicky & Norell, 2006:40)
ORDERED

120. Quadratojugal, shape of contact with jugal:
(0) – undivided
(1) – bifid around posterior end of jugal
(Modified from Farke et al., 2006:136)

121. Quadratojugal, contact with squamosal:
(0) – absent
(1) – present
(Brown and Schlaikjer, 1940a; Sampson et al., 2010:51)

122. Laterotemporal fenestra, position:
(0) – extends dorsal to ventral margin of orbit
(1) – positioned entirely below ventral limit of orbit
(Brown and Schlaikjer, 1940a; Forster, 1990)

123. Laterotemporal fenestra, size:
(0) – huge, subequal in size to orbit with long axis at least 50% basal skull length
(1) – greatly reduced, much smaller than orbit, no more than 30% skull length
(Modified after Brown and Schlaikjer, 1940a; Forster, 1990; Makovicky and Norell, 2006:35)

Frontal

124. Frontal, contribution to orbital margin:
(0) – present
(1) – absent
(Brown & Schlaikjer, 1940a; Sampson et al., 2010:53)

125. Frontal, contribution to dorsotemporal fenestra:
(0) – present
(1) – absent
(Lambe, 1915; Sampson et al., 2010:54)

126. Frontal, dorsocranial cavity complex:
(0) – absent
(1) – present, shallow depression
(2) – present, rimmed
(Modified from Forster 1990:3; modified from Makovicky and Norell, 2006:51; modified from Sampson et al., 2010:55; modified from Farke et al., 2011:38)
ORDERED

127. Frontal fontanelle, shape:
(0) – present, transversely narrow, slit-like
(1) – present, key-hole shaped, circular or elongate oval
(Modified from Forster 1990:49,50; Forster, 1996a:3; Sampson et al., 2010:56; Farke et al., 2011:39)

Squamosal

128. Squamosal-quadrate contact:
(0) – socket-like cotylus on ventrolateral squamosal for ball-like quadrate head
(1) – elongate groove on medial surface of squamosal to receive lamina of quadrate
(Hatcher et al., 1907; Forster, 1990; Sampson et al., 2010:63; Farke et al., 2011:43)

129. Squamosal, shape of temporal process:
(0) – simple
(1) – deeply bifurcate around temporal process of postorbital
(Modified from Makovicky and Norell, 2006:37)

130. Squamosal, parietosquamosal frill posterior expansion:
(0) – very slight, short shelf subequal to posterior extent of occipital condyle
(1) – intermediate shelf, expands posterior to the occipital condyle
(2) – extensive shelf, squamosal and parietal expanded well beyond the posterior extent of the quadrates forming true frill
(New Character; modified to some extent from Makovicky and Norell, 2006:52)
ORDERED

131. Squamosal, shape of posterior expansion:
(0) – sub-rectangular in outline
(1) – fan-shaped, sub-triangular
(2) – sickle-shaped, posteriorly narrowed
(Modified from Makovicky and Norell, 2006:36; Sampson et al., 2010:59, Farke et al., 2011:41)

132. Squamosal, posterior otic notch formed by the squamosal:
(0) – absent
(1) – present
(New Character)

133. Squamosal, shape of posterior edge of otic notch formed by the squamosal:
(0) – curved shape of anterior edge of squamosal transitioning into lateral squamosal
(1) – more than 90 angle between anterior edge squamosal and lateral squamosal
(2) – acute right angle
(3) – less than 90 angle
(New Character)

134. Squamosal, length relative to parietal on frill:
(0) – squamosal is longer than parietal and forms part of the posterior margin of the frill
(1) – squamosal shorter than parietal with part of the posterolateral portion of the frill made up of parietal
(Modified from Sampson et al., 2010:60-61)

135. Squamosal, medial lamina forming the posteriolateral floor of dorsotemporal fossa creating a pocket in the squamosal in dorsal view:
(0) – absent
(1) – present
(Modified from Dodson, 1986; Sampson et al., 2010:60, Farke et al., 2011:42)

136. Squamosal, posterior edge of squamosal is stepped so that lateral portion extends further posteriorly in dorsal view creating a "stepped margin":
(0) – absent
(1) – present
(Modified from Dodson, 1986; Sampson et al., 2010:60)

137. Squamosal, extent of step "stepped margin":
(0) – slight step where posterior expansion of step is less than the dorsoventral thickness of the pocket
(1) – posteriorly expanded to greater than twice the dorsoventral thickness of the pocket
(New Character)

138. Squamosal, thickened, rounded swelling along medial margin:
(0) – absent, lateral surface of squamosal flat to slightly convex
(1) – present, lateral surface of squamosal slightly concave
(Forster, 1990; Farke, 2005; Sampson et al., 2010:64, Farke et al., 2011:44)

139. Squamosals, orientation of squamosal (temporal bars):
(0) – parallel
(1) – posteriorly divergent
(Modified from Makovicky and Norell, 2006:39)

140. Squamosal, shape of dorsal edge of temporal bar as it meets posterior frill margin:
(0) – curves medially at the posterior end, arcing confluently into posterior frill margin
(1) – frill at acute angle
(Modified from Makovicky and Norell, 2006:53)

141. Squamosals, orientation of posterior edge:
(0) – angled anteromedially
(1) – angled posteromedially, squamosal contributing lateral portion of frill margin
(Modified from Makovicky and Norell, 2006:38)

142. Squamosal, ridge along the posterioventral edge of squamosal:
(0) – absent
(1) – present
(Modified from Farke et al., 2014:141)

143. Squamosal, dorsal otic ridge on lateral surface of the squamosal above otic notch:
(0) – absent
(1) – present
(New Character)

144. Squamosal, oblique dorsal ridge or bumps forming ridge along the dorsal surface of the squamosal:
(0) – absent
(1) – bumps present
(2) – well-developed pronounced peaked ridge
(Modified from Evans and Ryan, 2015:98)
ORDERED

Parietal

145. Parietal, anterior extent on dorsum of skull relative to occipital condyle:
(0) – anterior end of parietal located well in front of occipital condyle
(1) – anterior end of parietal lies directly over occipital condyle
(Sampson et al., 2010:57; Farke et al., 2011:40)

146. Parietal, shape of anterior skull roof:
(0) – flat or gently convex
(1) – sharp midline crest
(Modified from Farke et al., 2014:138)

147. Parietosquamosal contact, shape in dorsal view:
(0) – lancelet or straight
(1) – curved, medially concave
(Modified from Forster 1990:66; Sampson et al., 2010:57; Farke et al., 2011:45)

148. Parietal, concave median embayment on posterior margin:
(0) – absent
(1) – present
(Makovicky and Norell, 2006:55; Sampson et al., 2010:66; Farke et al., 2011:46)

149. Parietal, shape of concave median embayment on posterior edge of parietal:
(0) – shallow, may include the whole back of the frill
(1) – restricted to center of margin, incipient
(2) – restricted to center of margin, deep notch-like
(Modified from Forster et al., 1993:9; Forster, 1990:83; Sampson et al., 2010:67, Farke et al., 2011:47)

150. Parietal, location of posterior-most point of parietal:
(0) – on midline
(1) – between midline and lateral-most corner
(2) – at lateral-most corner adjacent to squamosal
(Modified from Holmes et al., 2001:18; Sampson et al., 2010:68)

151. Parietosquamosal frill, elongation into frill:
(0) – absent
(1) – present elongation of parietal onto frill

152. Parietosquamosal frill, length relative to basal skull length:
(0) – short, 60% or less
(1) – elongate, more than 70%
(Modified from Hatcher et al., 1907; Lehman, 1990; Forster, 1990: XX; Sampson et al., 2010:69)

153. Parietosquamosal frill, location of maximum transverse width true frill:
(0) – anteriorly, in association with proximal third of frill
(1) – widest part in middle, frill round
(2) – lateral edges roughly parallel
(3) – posteriorly
(Modified from Holmes et al., 2001:17; Sampson et al., 2010:70)

154. Parietal, dorsotemporal channels on anterior parietal (parietal sulci):
(0) – absent
(1) – present or secondarily roofed
(Marsh, 1892; Sampson et al., 2010:71)

155. Parietal, overall shape in lateral view:
(0) – nearly straight along midline in lateral view and gently arched from side to side
(1) – "saddle-shaped," dorsally concave in lateral view with upturned posterior margin, and arched strongly from side to side
(Sampson et al., 2010:72)

156. Parietal, sharp median crest:
(0) – present as ridge or line
(1) – absent
(Sampson et al., 1997; Sampson et al., 2010:75; Farke et al., 2011:49)

157. Parietal fenestra:
(0) – absent, no fenestra
(1) – present
(Modified from Forster, 1990; Makovicky and Norell, 2006:54; Sampson et al., 2010:73)

158. Parietal fenestra, orientation:
(0) – long axis directed transversely
(1) – long axis directed axially
(2) – axial and transverse axes equal
(Modified from Forster, 1990; Sampson et al., 2010:80)

159. Parietal fenestra size, maximum proximodistal diameter:
(0) – large, greater than 45% total parietal length
(1) – tiny, 35% or less total parietal length
(Modified from Forster et al., 1993:8; Sampson et al., 2010:81)

160. Parietal, rim on anterior medial margin of dorsotemporal fenestra:
(0) – absent
(1) – present, well-defined but narrow laterally projecting rim defines medial margin of fenestra
(2) – present, well-defined and wide laterally projecting rim defines medial margin of fenestra
(Modified from Forster, 1990: XX; Sampson et al., 2010:74; Farke et al., 2011:48)

161. Parietal, anteroposterior thickness of transverse bar at narrowest point:
(0) – narrow and straplike, less than 10% total parietal length
(1) – broad, 20% or more of total parietal length
(Modified from Forster et al., 1993:3; Sampson et al., 2010:76; Farke et al., 2011:50)

162. Parietal, cross-sectional shape of transverse bar:
(0) – flat
(1) – round
(New Character)

163. Parietal, relative anteroposterior depth of transverse bar:
(0) – subequal medial to lateral
(1) – tapering medially
(Sampson et al., 2010:77)

164. Parietal, cross-sectional shape of median bar:
(0) – flat to subtriangular
(1) – round to lenticular
(2) – V-shaped without thickening
(Modified from Holmes et al., 2001:24; Sampson et al., 2010:78)

165. Parietal, median bar, transverse width:
(0) – narrow and strap-like, transverse width less than 10% total parietal length
(1) – relatively wide, transverse width 15% or more of total parietal length
(Holmes et al., 2001:23; Sampson et al., 2010:79; Farke et al., 2011:51)

Braincase

166. Supraoccipital, contribution to foramen magnum:
(0) – forms dorsal margin of foramen magnum
(1) – eliminated from margin by exoccipital-exoccipital contact on midline
(Brown & Schlaikjer, 1940a; Forster 1990:63; Makovicky and Norell, 2006:65; Farke et al., 2011:69)

167. Supraoccipital, ventrolateral processes:
(0) – absent
(1) – present
(Brown & Schlaikjer, 1940a)

168. Supraoccipital, ventrolateral processes, dorsolateral ridge between midline supraoccipital ridge and top of exoccipital wing:
(0) – absent
(1) – present
(New Character)

169. Supraoccipital, ventrolateral processes, dorsolateral ridge orientation:
(0) – oriented at roughly 45 degrees
(1) – originate at 45 degrees and then extend dorsally parallel to the midline ridge
(New Character)

170. Supraoccipital, deep fossa between dorsolateral strut and exoccipital:
(0) – no fossa
(1) – shallow depression
(2) – deep fossa that forms dorsomedially inclined strut that joins dorsolateral strut
(New Character)
ORDERED

171. Supraoccipital orientation:
(0) – anteriorly inclined relative to basioccipital
(1) – posteriorly inclined or in same plane as posterior face of basioccipital
(Makovicky & Norell, 2006:66)

172. Supraoccipital shape:
(0) – tall, triangular
(1) – or wider than tall, trapezoid
(2) – or square
(Makovicky & Norell, 2006:67)

173. Supraoccipital, midline vertical ridge:
(0) – absent
(1) – present
(New Character)

174. **Supraoccipital, midline vertical ridge, form:**
(0) – rounded ridge
(1) – ridge forms fin
(New Character)

175. **Supraoccipital, shape of posterior surface:**
(0) – flat, convex, or with midline ridge
(1) – midline depression along base of midline ridge
(Modified from Farke et al., 2014:142)

176. **Basioccipital, contribution to occipital condyle:**
(0) – forms more than 2/3 of occipital condyle
(1) – forms less than 1/3 of the occipital condyle
(Brown & Schlaikjer, 1940a)

177. **Basioccipital, participates in foramen magnum:**
(0) – present
(1) – or basioccipital is excluded from foramen magnum
(Makovicky & Norell, 2006:57)

178. **Basioccipital, subcondylar region form:**
(0) – raised ridge ventral to occipital condyle
(1) – flat or rounded
(New Character)

179. **Basioccipital, presence of fossa in raised subcondylar region:**
(0) – absent
(1) – fossa is present ventral to occipital condyle
(New Character)

180. **Basioccipital, subcondylar region, ventral process lateral to midline vertical ridge:**
(0) – present between ventral process and basitubera
(1) – absent
(New Character)

181. **Basioccipital, contribution to basal tubera:**
(0) – excluded from basal tubera by basisphenoid and limited to occipital midline
(1) – basioccipital contributes to basal tubera present
(Modified from Makovicky and Norell, 2006:58)

182. **Basal tubera, position:**
(0) – flat, in plane with basioccipital plate
(1) – everted posterolaterally, forming lip beneath occipital condyle
(Modified from Makovicky and Norell, 2006:60)

183. **Basal tubera, fossa on posterior surface:**
(0) – absent
(1) – present
(New Character)

184. **Basisphenoid, notch between posteroverntral edge of basisphenoid and base of basipterygoid process:**
(0) – deep
(1) – shallow and base of basipterygoid process close to basioccipital tubera
(Modified from Makovicky and Norell, 2006:61)

185. **Foramen magnum, visible in posterior view:**
(0) – visible in posterior view
(1) – dorsally oriented and obscured by occipital condyle
(New Character)

186. **Olfactory nerve (I), frontal contribution to exit from braincase:**
(0) – present, frontal forms roof of olfactory tract
(1) – absent, olfactory tract enclosed entirely by ossification of the laterspenoid.
(Forster, 1990)

187. **Exoccipital, exits for cranial nerves in exoccipital:**
(0) – three foramina
(1) – two foramina
(Brown & Schlaikjer, 1940a; Forster, 1990; Makovicky and Norell, 2006:62)

188. Exoccipital–quadrate:
(0) – completely separated by ventral flange of squamosal
(1) – or in contact
(Makovicky & Norell, 2006:63)

189. Paroccipital process, dorsoventral distal expansion:
(0) – distal process only slightly expanded
(1) – distal process expanded to at least .8 to two times the depth at its narrowest point
(Brown & Schlaikjer, 1940a; Makovicky and Norell, 2006:64)

Quadrate

190. Quadrate, shape of the anterior quadrate shaft in lateral view:
(0) – convex
(1) – straight
(Modified from Makovicky and Norell, 2006:41)

Palatine and vomer

191. Secondary palate, relative contribution of maxilla:
(0) – maxilla forms at least 45% of the length of the secondary palate
(1) – maxilla contributes only to the posterior portion, forms 30% or less of secondary palate
(Brown & Schlaikjer, 1940a)

192. Palatine, shape and relationship to maxilla:
(0) – palatine contacts nearly the entire medial surface of the maxilla, restricting size of choanae, anterodorsal process embraces posterior end of vomer
(1) – palatine contacts only the posterior one-third of medial surface of maxilla, contact with vomer lost, choanae enlarged
(Osmólska, 1986)

193. Elongate parasagittal process of the palatine:
(0) – absent
(1) – present
(Makovicky & Norell, 2006:42)

194. Vomer, relationship to maxillae on secondary palate:
(0) – vomers insert between maxillae at the rear of the secondary palate
(1) – vomers meet posterior margin of maxillae on secondary palate, do not insert between maxillae, so that the maxillae contact each other anterior to the choanae in palatal view and obscure the tip of the vomer
(Modified from Makovicky & Norell, 2006:21)

Pterygoid

195. Pterygoid, contacts maxilla posterior to the tooth row:
(0) – absent
(1) – present
(Makovicky & Norell, 2006:47)

196. Pterygoid, mandibular process:
(0) – long, extending well below maxillary tooth row
(1) – short
(Modified after Makovicky & Norell, 2006:49)

197. Pterygoid, mandibular process makeup:
(0) – jointly formed by pterygoid and ectopterygoid
(1) – formed only by pterygoid
(Modified from Makovicky and Norell, 2006:50)

198. Pterygoid, ventral ridge on mandibular process of pterygoid defining Eustachian canal:
(0) – absent
(1) – present
(Makovicky and Norell, 2006:46)

199. Pterygoid, prominent posterior midline process:

(0) – absent
(1) – present
(Modified from Makovicky and Norell, 2006:48)

Ectopterygoid

200. Ectopterygoid contacts jugal:
(0) – present
(1) – or ectopterygoid reduced and restricted to contact with maxilla
(Brown & Schlaikjer, 1940a; Makovicky & Norell, 2006:44)

201. Ectopterygoid visible in palatal view:
(0) – exposed
(1) – or reduced and concealed in palatal view
(Makovicky & Norell, 2006:43)

Mandible

202. Lower jaw, level of mandibular articulation:
(0) – at or slightly below occlusal surface of tooth row
(1) – depressed well below level of occlusal surface of tooth row
(Ostrom, 1964; Forster, 1990; Sampson et al., 2010:112)

203. External mandibular fenestra:
(0) – present
(1) – absent
(New Character)

Predentary

204. Predentary/dentary anteroposterior ratio:
(0) – less than two thirds of dentary length
(1) – or equal to or more than two-thirds of dentary length
(Makovicky & Norell, 2006:70; Sampson et al., 2010:113)

205. Predentary shape in occlusal view:
(0) – broad and rounded anteriorly, "U" shaped
(1) – acute, "V"-shaped
(New Character)

206. Predentary, shape of dorsal surface:
(0) – shallow or flat
(1) – scoop-like
(Modified from Makovicky and Norell, 2006:68)

207. Predentary, dentary processes:
(0) – ventral processes much longer than abbreviated dorsal processes
(1) – dorsal and ventral processes elongate and subequal in length
(Sampson et al., 2010:114; Farke et al., 2011:70)

208. Predentary, ventral dentary process shape:
(0) – wedge shaped and mediolaterally broad
(1) – splint shaped
(Modified from Makovicky and Norell, 2006:69)

209. Predentary, ventral dentary process bifurcation:
(0) – absent
(1) – present
(New Character)

210. Predentary, occlusal margin:
(0) – mediolaterally thin
(1) – thickened mediolaterally
(New Character)

211. Predentary buccal margin:
(0) – with round beveled edge
(1) – or with grooved, triturating edge
(Makovicky & Norell, 2006:71)

212. Predentary, orientation of triturating surface:
(0) – nearly horizontal
(1) – inclined steeply laterally
(2) – inclined medially
(Lehman, 1990; Forster, 1990; Dodson et al., 2004:57; Farke et al., 2011:71)

213. Predentary, contact of the lateral process with dentary:
(0) – smooth
(1) – grooved dorsally
(2) – bears large pit
(Modified from Makovicky and Norell, 2006:72)

Dentary

214. Dentary, symphyseal area:
(0) – small excluding splenial
(1) – large, forming strong immobile bond with participation of splenial
(Modified from Makovicky and Norell, 2006:73)

215. Dentary, presence of diastema at anterior end:
(0) – absent
(1) – present
(Modified from Makovicky and Norell, 2006:74)

216. Dentary, presence of ventral flange:
(0) – absent
(1) – present along ventral edge
(Modified from Makovicky and Norell, 2006:76)

217. Dentary, lateral ridge confluent with cutting surface of predentary:
(0) – absent or faintly present
(1) – present as buchal crest
(Farke et al., 2011:72)

218. Dentary, prominent medial expansion of cheeks of the central mandible in the middle of the tooth row formed by wide Meckelian groove separating tooth-bearing part of the jaw from external surface:
(0) – present
(1) – absent
(Modified from Makovicky and Norell, 2006:77)

219. Dentary, shape of area lateral to tooth row:
(0) – smooth
(1) – rugose and sculpted
(Modified from Makovicky and Norell, 2006:78)

220. Dentary anteroventral fossa below lateral ridge:
(0) – absent
(1) – present
(New Character)

221. Dentary anteroventral fossa below lateral ridge size:
(0) – deep
(1) – weakly present
(New Character)

222. Dentary, shape of ventral margin in adults:
(0) – strongly convexly bowed
(1) – straight
(Brown and Schlaikjer, 1940a; Forster, 1990:73; Makovicky and Norell, 2006:75; Sampson et al., 2010:117)

223. **Dentary, posterior extent of tooth row:**
(0) – terminates anterior to the center of the coronoid process
(1) – terminates even with the coronoid process
(2) – terminates posterior to the coronoid process
(Brown & Schlaikjer, 1940a; Chinnery and Weishampel 1998; modified from Makovicky and Norell, 2006:82; Sampson et al., 2010:118; Farke et al., 2011:73)
ORDERED

224. **Dentary, overall shape of coronoid process:**
(0) – short, with gently convex apex, base of ascending ramus anteroposteriorly expanded
(1) – tall, expanded at apex into anteriorly projecting hook, base of ascending ramus anteroposteriorly restricted
(Lull, 1933; Sampson et al., 2010:119)

225. **Dentary, shape of dorsal end of coronoid process:**
(0) – rounded
(1) – anterior expansion
(Modified from Makovicky and Norell, 2006:81)

226. **Dentary, coronoid process notch along posterior edge of dentary:**
(0) – absent
(1) – present
(Modified from Farke et al., 2014:145)

227. **Dentary, width of coronoid process notch:**
(0) – wide
(1) – constricted notch
(Modified from Farke et al., 2014:146)

228. **Dentary, separation of body from ascending ramus of coronoid process:**
(0) – absent
(1) – present
(Granger & Gregory, 1923; Sampson et al., 2010:120)

Splenial

229. **Splenial, shape:**
(0) – nearly as deep as the body of the dentary, does not contact articular, angular exposed in medial view
(1) – shallow, contacts articular, covers angular in medial view
(Brown & Schlaikjer, 1940a, 1940b; Sampson et al., 2010:121)

230. **Posterior end of splenial:**
(0) – simple or with shallow dent
(1) – or withdeep bifid overlap of angular
(Makovicky & Norell, 2006:80)

Coronoid

231. **Coronoid bone, size:**
(0) – large and lobate, with expanded dorsal end much deeper than ventral that inserts between the dentary and splenial to contact, contacts the splenial
(1) – small and strap-like with subequal depth throughout, no contact with splenial
(New Character derived from Brown & Schlaikjer, 1940a, 1940b; modified from Makovicky and Norell, 2006:83)

Angular

232. **Angular, exposure in lateral view:**
(0) – extensive
(1) – greatly restricted
(New Character derived from Brown & Schlaikjer, 1940a)

233. Angular, shape of lateral surface:
(0) – or flat or slightly convex
(1) – angular bears a raised emargination along posteroventral margin of mandible, lateral surface distinctly concave
(Modified after Makovicky & Norell, 2006:88)

234. Angular, texture of lateral surface:
(0) – smooth
(1) – strongly sculptured
(Modified from Farke et al., 2014:135)

235. Angular, presence of one or more small lateral tubercles along ventral rib below glenoid articulation:
(0) – absent
(1) – present
(Modified from Makovicky and Norell, 2006:87)

Surangular

236. Surangular dorsally projecting ridge on lateral margin:
(0) – present
(1) – absent
(Makovicky & Norell, 2006:84)

237. Surangular, lateral surface:
(0) – flat or only weakly convex
(1) – with pronounced laterally convex curvature (in the transverse plane) between the coronoid process and glenoid region
(Modified from Makovicky and Norell, 2006:85)

238. Surangular, lateral process below glenoid:
(0) – absent
(1) – present; surangular knob
(Farke et al., 2014:139)

239. Surangular, presence of tab forming lateral wall to glenoid cotyle:
(0) – absent
(1) – present
(Modified from Makovicky and Norell, 2006:86)

240. Angular–surangular–dentary contact:
(0) – triradiate
(1) – surangular with long ventral process overlapping angular, dentary-surangular and angular-surangular sutures form acute angle on lateral face of mandible
(Makovicky & Norell, 2006:89)

Prearticular

241. Prearticular-dentary contact:
(0) – absent
(1) – present
(Brown & Schlaikjer, 1940a, 1940b; Makovicky and Norell, 2006:79; Sampson et al., 2010:122)

Articular

242. Articular, size and contribution to mandibular glenoid:
(0) – articular forms only the medial 1/3 of the articular surface, extends posteriorly as far as the angular
(1) – articular enlarged, forms half of the articular surface, extends behind angular to form rear of lower jaw
(New Character derived from Brown & Schlaikjer, 1940a)

243. Articular, mandibular glenoid in dorsal view:
(0) – narrow and flush with medial margin of surangular flange
(1) – glenoid region medially expanded and forming lingual process
(Modified from Makovicky and Norell, 2006:90)

244. **Glenoid, surface of prearticular and articular below glenoid:**
(0) – smooth
(1) – wide, semicircular ventral process near medial face of glenoid
(Modified from Makovicky and Norell, 2006:91)
245. **Articular, retroarticular process length:**
(0) – long
(1) – short or absent
(Modified from Makovicky and Norell, 2006:92)

Dentition

246. **Tooth, number of roots:**
(0) – one
(1) – two
(Forster 1990:34; Makovicky and Norell, 2006:95; Sampson et al., 2010:123; Farke et al., 2011:74)
247. **Teeth, presence of median primary ridge:**
(0) – absent
(1) – very weak and wide median ridge on at least some maxillary teeth
(2) – all maxillary and dentary teeth with distinct primary ridge
(Modified from Makovicky and Norell, 2006:98)
ORDERED
248. **Teeth, presence of cingulum:**
(0) – absent
(1) – present
(Modified from Makovicky and Norell, 2006:100)
249. **Teeth, relationship of the base of primary ridge and the cingulum:**
(0) – confluent with the cingulum on maxillary teeth
(1) – base of primary ridge set back from cingulum; forms a continuous ridge at the crown base
(Modified from Makovicky and Norell, 2006:99)
250. **Teeth, enamel on lingual and buccal surfaces of teeth:**
(0) – covered with enamel
(1) – enamel restricted to lateral side of maxillary and medial side of dentary teeth
(Modified from Makovicky and Norell, 2006:102)
251. **Tooth crown height in mid tooth row position:**
(0) – subequal to tooth crown width
(1) – taller than wide
(New Character)
252. **Teeth, crown shape in lateral view:**
(0) – radiate or pennate
(1) – ovate
(Modified from Makovicky and Norell, 2006:106)
253. **Cheek teeth, shape of roots:**
(0) – with cylindrical roots
(1) – roots with mesial and distal faces flattened to slightly grooved
(Modified from Makovicky and Norell, 2006:105)
254. **Teeth, cheek teeth spacing:**
(0) – spaced
(1) – closely appressed
(Makovicky & Norell, 2006:96)
255. **Teeth, occlusion:**
(0) – occlude at an oblique angle
(1) – at a vertical angle
(2) – at a vertical angle, dentary teeth have a horizontal shelf on the labial face (leptoceratopsid condition)
(Makovicky & Norell, 2006:97)

256. Tooth, number of replacements per alveolus:
(0) – one or two replacement teeth
(1) – three or more replacement teeth
(Sereno 1999:137; Makovicky and Norell, 2006:101; Farke et al., 2011:76)

257. Tooth magazine, case-like alveolar slots for vertical tooth families formed by spongy bone:
(0) – absent
(1) – present
(Brown & Schlaijker, 1940a)

258. Premaxillary teeth:
(0) – absent
(1) – present
(New Character)

259. Premaxillary teeth:
(0) – three or more teeth in premaxilla
(1) –two or one tooth in premaxilla
(Makovicky & Norell, 2006:93)

260. Premaxillary teeth morphology:
(0) – with carinae and in some cases serrations
(1) – premaxillary teeth peglike, crown without carinae
(Makovicky & Norell, 2006:94)

261. Maxillary teeth, number of alveoli in maxilla:
(0) – 11 or less
(1) – more than 12 and less than 14
(2) – more than 15 and less than 18
(3) – more than 20 and less than 29
(4) – more than 30
(New Character)
ORDERED

262. Dentary teeth, number of alveoli in dentary:
(0) – 11 or less
(1) – more than 12 and less than 18
(2) – more than 19 and less than 29
(3) – more than 30
(Modified from Makovicky & Norell, 2006:104; Farke et al., 2011:75)
ORDERED

263. Dentary teeth, shape of tooth crowns:
(0) – continuous, smooth root crown transition
(1) – bulbous expansion at root-crown transition on labial side of tooth, sometimes worn to form notch or shelf
(Modified from Makovicky and Norell, 2006:103)

Axial Skeleton

264. Atlantal intercentrum, shape:
(0) – semicircular
(1) – disc shaped
(Modified from Makovicky and Norell, 2006:107)

265. Atlantal intercentrum, fusion to odontoid:
(0) – absent
(1) – present
(Modified from Makovicky and Norell, 2006:108)

266. Atlantal intercentrum, fusion to atlantal neuropophyses:
(0) – absent
(1) – present
(Modified from Makovicky and Norell, 2006:109)

267. Cervical vertebrae, formation of syncervical:
(0) – absent
(1) – present
(Modified from Forster 1990:122; Sampson et al., 2010:127; Farke et al., 2011:77)

268. Cervical vertebrae, form of syncervical:
(0) – C1-3 fused or tightly articulated, atlantal intercentrum present as a ventrally placed, wedge-like bone
(1) – C1-3 firmly fused, atlantal intercentrum (= hypocentrum) forms a complete ring
(Modified from Forster 1990:122; Sampson et al., 2010:127; Farke et al., 2011:77)

269. Axis, neural spine shape and orientation
(0) – blade-like and nearly vertical, overhangs only anterior-most portion of C3
(1) – blade-like morphology lost, spine steeply angled to reach posterior margin of C3
(Brown & Schlaikjer, 1940a; Sereno 1999:141; modified from Makovicky and Norell, 2006:110, 111; Sampson et al., 128: Farke et al., 2011:78)

270. Atlantal rib:
(0) – present
(1) – absent
(Brown & Schlaikjer, 1940a; Sampson et al., 2010:129; Farke et al., 2011:79)

271. Cervical centra, presence of ventral keels:
(0) – ventral keels present
(1) – some or all postaxial centra without keels
(Modified from Farke et al 2014:147)

272. Cervical rib shape:
(0) – L or Y-shaped elongate spline of bone with bifurcated end
(1) – x-shaped accessory dorsal process present (tuberculum of Xu et al 2010)
(New Character)

273. Mid-dorsal neural spine height:
(0) – subequal in height to centrum height
(1) – 2 times the centrum height
(2) – three times centrum height
(New Character)

274. Dorsal vertebrae, zygapophyseal articular surfaces shape:
(0) – with flat articulations on zygapophyses
(1) – tongue and grooves articulations on zygapophyses
(Makovicky & Norell, 2006:113)

275. Dorsal vertebrae, length of centra:
(0) – relatively axially elongate, lengths exceed width length/height of centra 1.2 or more
(1) – axially shortened, width/height exceeds length of centra 0.9 or less
(Modified from Sampson et al., 2010:130; Farke et al., 2011:80)

276. Dorsal vertebrae, elongation of tallest neural arches:
(0) – arch shorter than centrum
(1) – present, arch height taller than centrum
(New Character)

277. Dorsal vertebrae, posteriormost dorsals, zygopohyseal facets horizontal, zygopophyses coalesce:
(0) – absent
(1) – present
(New Character)

278. Sacrum, number of vertebrae:
(0) – five
(1) – six
(2) – seven
(3) – eight or more
(Modified from Makovicky and Norell, 2006:114)

279. Sacrum, longitudinal sulcus on ventral surface:
(0) – absent
(1) – present restricted to caudal sacral vertebrae
(2) – deep across nearly all sacral vertebrae
(Modified from Lambe, 1915; Lehman, 1989, 1990; Sampson et al., 2010:131; Farke et al., 2011:81)
ORDERED

280. Sacrum, shape in dorsal view:
(0) – rectangular
(1) – suboval, mid-sacral ribs longest
(Modified from Makovicky and Norell, 2006:115)

281. Sacrum, ventral expansion of 1st sacral rib to attach along entire lateral side of vertebra:
(0) – absent
(1) – present
(New Character derived from Lull, 1933; Lehman, 1990)

282. Anterior caudal neural spines, height:
(0) – short
(1) – tall twice centrum height
(Modified from Makovicky & Norell, 2006:116)

283. Anterior caudal neural spines, inclination:
(0) – anteriorly inclined
(1) – erect
(Modified from Makovicky & Norell, 2006:116)

284. Mid caudal neural spines, height:
(0) – less than or equal to 2 times centrum height
(1) – 2.1 to 3 times centrum height
(2) – 3.1 to 4 times centrum height
(Modified from Farke et al., 2014:140)

285. Distal caudals, tail terminates with:
(0) – series of cylindrical caudals that are devoid of neural spines and chevrons
(1) – neural spines and chevrons persist virtually to the end of tail
(Modified from Makovicky and Norell, 2006:117)

286. Caudal chevrons, shape of distal caudal chevrons:
(0) – lobate expanded shape
(1) – rod-like
(Modified from Makovicky and Norell, 2006:118)

Pectoral Girdle and Limb

287. Scapula, shape in lateral view:
(0) – distinctly curved in lateral view
(1) – relatively straight
(Makovicky & Norell, 2006:120)

288. Scapula, position of blade relative to glenoid:
(0) – at acute angle
(1) – almost perpendicular to glenoid
(Modified from Makovicky and Norell, 2006:121)

289. Scapula, relative contribution to glenoid fossa:
(0) – scapula and coracoid contribute equally
(1) – scapula contributes well over half of the glenoid
(Brown & Schlaikjer, 1940a; Sereno 1999:145; Farke et al., 2011:82)

290. Glenoid fossa, size and shape:
(0) – glenoid relatively short, unexpanded mediolaterally
(1) – glenoid expanded anteroposteriorly and expanded medially
(Chinnery, 2003)

291. Glenoid fossa, position of humeral head:
(0) – positioned medially in the glenoid fossa
(1) – positioned in the lateral portion of the glenoid fossa
(New Character)

292. Scapular ridge or scapular spine:
(0) – absent
(1) – present
(New Character)

293. Scapula, orientation of scapular spine:
(0) – runs obliquely across blade
(1) – runs longitudinally along blade
(Sampson et al., 2010: 133)

294. Scapula, size of acromion process:
(0) – small, restricted to proximal end
(1) – enlarged, extends distal to the glenoid fossa
(Modified from Makovicky and Norell, 2006:123; Maidment and Barrett, 2011)

295. Coracoid, shape of anterior blade:
(0) – smooth, arcuate anterior portion
(1) – bearing large anterolateral ridge near confluence of anterior and ventral margins
(Modified from Makovicky and Norell, 2006:122)

296. Coracoid, medial opening of coracoid foramen connected to coraco-scapular suture by sulcus:
(0) – absent
(1) – present
(New Character)

297. Coracoid, concave inflection between coraco-scapular suture and anterior margin of coracoid:
(0) – absent
(1) – present
(New Character)

298. Humerus, length of deltopectoral crest in adult:
(0) – short, less than 40% total humeral length
(1) – elongate, subequal to or greater than 45% of the total humeral length
(Chinnery, 2001, 2004)

299. Humerus, shape of margin of deltopectoral crest:
(0) – concave
(1) – straight to convex
(Maidment and Barrett, 2011)

300. Humerus, triceps fossa on posteriolateral deltopectoral crest:
(0) – shallow with poorly defined margins
(1) – deep with well-defined margins
(Maidment and Barrett, 2011)

301. Humerus, position of foramen on posteriomedial humerus relative to apex of deltopectoral crest:
(0) – level with
(1) – proximal to
(Maidment and Barrett, 2011)

302. Humerus, orientation of deltopectoral crest:
(0) – projects anteriorly
(1) – projects anterolateraly
(Maidment and Barrett, 2011)

303. Radius, lateral and medial tuberosities along distal half of shaft:
(0) – absent
(1) – present
(Modified from Makovicky and Norell, 2006:144)

304. Distal carpals:
(0) – more than two distal carpals
(1) – less than two distal carpals
(Makovicky & Norell, 2006:124)

305. Manus and pes size:
(0) – manus much smaller than pes
(1) – closer to pes in size
(Makovicky & Norell, 2006:125)

306. Manus number of metatarsal digits:
(0) – 5
(1) – 4 or less
(New Character)

307. Manal unguals, shape:
(0) – mediolaterally compressed, taper to distal tip
(1) – dorsoventrally compressed, blunt or rounded distal tip
(Brown, 1914b; Chinnery & Weishampel, 1998:64; Sampson et al, 2010:136; Farke et al., 2011:85)

308. Manal and pedal penultimate phalanges, shape:
(0) – length exceeds width
(1) – width exceeds length
(Sampson et al., 2010:137; Farke et al., 2011:86)

309. Manus, relative lengths in digit three:
(0) – articulated phalanges exceed MCIII in length
(1) – articulated phalanges shorter than MCIII in length
(New Character)

310. Manal and pedal phalanges, flexor pits:
(0) – present
(1) – absent
(New Character)

Pelvic Girdle and Limb

311. Ilium, lateral eversion of dorsal margin:
(0) – absent
(1) – present
(Hatcher et al., 1907; Forster 1990: 108 & 109; Chinnery, 2001, 2004; Sampson et al., 2010:138; Farke et al., 2011:87)

312. Ilium, relative lengths of pubic and ischial peduncles:
(0) – pubic and ischial peduncles long, extend well below body of ilium approximately the same distance
(1) – ischial peduncle reduced along ventral aspect, pubic peduncle projects further ventrally than ischial peduncle
(Sampson et al., 2010:139; Farke et al., 2011:88)

313. Pubis, prepubic process:
(0) – short and unexpanded distally
(1) – elongate, distal end greatly expanded dorsoventrally
(Forster 1990:111; Makovicky and Norell, 2006:128; Sampson et al., 2010:140; Farke et al., 2011:89)

314. Pubis, position and length of postpubic rod:
(0) – relatively long but extends past ischial peduncle of ilium, arises ventral to acetabulum and lies along ventral and ventromedial margin of ischium
(1) – very abbreviated, terminates at level of ischial peduncle, arises medial to acetabulum and passes entirely medial to ischium
(Forster, 1990:110; Makovicky and Norell, 2006:127; Sampson et al., 2010:141; Farke et al., 2011:90)

315. Pubis and ischium, morphology of contributions to acetabulum:
(0) – pubic acetabular surface faces posteriolaterally, pubis and pubic process of ischium contribute equally to ventral margin of acetabulum
(1) – pubic acetabular surface faces laterally and forms a partial medial wall to the acetabulum, pubic process of ischium elongated and meets pubis close to anterior margin of acetabulum, ventral portion of pubic acetabular surface lies medial to pubic ramus of ischium
(Sampson et al., 2010:142; Farke et al., 2011:91)

316. Pubis, cross-sectional shape of postpubic shaft:
(0) – round
(1) – mediolaterally flattened, bladelike
(Modified from Makovicky and Norell, 2006:126)

317. Ischium, cross-sectional shape of shaft:
(0) – thick and ovoid
(1) – laterally compressed and bladelike, tapered dorsally
(Forster 1990:112; Sampson et al., 2010:143; Farke et al., 2011:92)

318. Ischium, orientation of shaft:
(0) – nearly straight or slightly decurved
(1) – broadly and continuously curved
(Brown & Schlaikjer, 1940a; Forster 1990:113; Makovicky and Norell, 2006:129; Sampson et al., 2010:144; Farke et al., 2011:93)

319. Femur, morphology of greater and lesser trochanters:
(0) – trochanters distinct and located below the level of the femoral head
(1) – trochanters coalesced and level with the femoral head
(Brown & Schlaikjer, 1940a; Dodson et al., 2004:72; Sampson et al., 2010:145; Farke et al., 2011:94)

320. Femur, size of fourth trochanter:
(0) – large and pendant
(1) – small, reduced to low prominence
(Brown & Schlaikjer, 1940a; Sereno 1999:154; Makovicky and Norell, 2006:130; Sampson et al., 2010:146; Farke et al., 2011:95)

321. Femur-tibia proportion:
(0) – tibia longer than, or equal to femur length
(1) – femur longer than tibia
(Brown & Schlaikjer, 1940a; Forster 1990:103; Makovicky and Norell, 2006: 131; Sampson et al., 2010:147; Farke et al., 2011:96)

322. Pes, metatarsal proportions:
(0) – length of MT I 80% the length of MT II
(1) – MT I reduced less than 70% the length of MT II
(Modified from Sampson et al., 2010:148; Farke et al., 2011:97)

323. Pes, shape:
(0) – gracile with long, constricted metatarsus, elongate phalanges
(1) – short and uncompressed, all phalanges wider than long
(Modified from Makovicky and Norell, 2006:132)

324. Pedal unguals, shape:
(0) – pointed
(1) – rounded, hooflike
(Modified from Makovicky and Norell, 2006:133)

Epiossifications

325. Marginal undulations on squamosal, often with dermal ossifications capping these undulations:
(0) – absent
(1) – present
(New Character)

326. Marginal undulations on parietal, often with dermal ossifications capping these undulations:
(0) – absent
(1) – present
(Modified from Forster 1990:91 & 92; Makovicky and Norell, 2006:56; Sampson et al., 2010:84; Farke et al., 2011:53)

327. Parietosquamosal frill, imbrication of squamosal undulations:
(0) – absent
(1) – present
(New Character)

328. Parietosquamosal frill, imbrication of parietal undulations:
(0) – absent
(1) – present
(New Character)

Episquamosals

329. Episquamosal, shape:
(0) – crescentic or ellipsoidal, often with constricted base
(1) – triangular/peaked often elongated
(Dodson et al., 2004:45; Sampson et al., 2010 87; Farke et al., 2011:54)

330. Episquamosals, large gaps between epiquamosals:
(0) – large gaps present between episquamosals
(1) – absent, episquamosals nearly touch one another along squamosal margin
(New Character)

331. Episquamosal, asymmetry:
(0) – all subequal in size
(1) – marked asymmetry in size
(Modified from Sampson et al., 2010:85, 86)

332. Episquamosal, enlargement of anterior episquamosal:
(0) – absent
(1) – present
(New Character)

333. Episquamosal, enlargement of posteriorly penultimate (es2) episquamosal:
(0) – absent
(1) – present
(New Character)

334. Episquamosal, enlargement of posteriormost (es1) episquamosal:
(0) – absent
(1) – present
(New Character)

335. Length and height of all episquamosals:
(0) – sub-equilateral delta or crescentic shaped
(1) – all epiquamosals long with high epiquamosal length to height ratio
(Sampson et al., 2010:85)

336. Number of episquamosals or nodess on squamosal:
(0) – two
(1) – three
(2) – four
(3) – five
(4) – six or seven episquamosals
(5) – eight or more episquamosals
(Modified from Farke et al., 2011:55)
ORDERED

Epiparietosquamosal

337. Marginal ossification crossing parietal-squamosal contact (location eps):
(0) – absent
(1) – present
(Modified from Dodson et al., 2004:43; Sampson et al., 2010:91; Farke et al., 2011:56)

Epiparietals

338. Epiparietal, fusion to adjacent epiparietals at base:
(0) – absent
(1) – present
(Modified from Holmes et al., 2001:29)

339. Epiparietal, presence of elongated spikes:
(0) – absent
(1) – one spike present
(2) – two spikes present
(3) – more than two spikes present
ORDERED

340. Epiparietal, flattening of spikes into elongated, flat, blade-like spike:
(0) – absent
(1) – present
(New Character)

341. Epiparietal, presence of more than 3 dorsally recurved epiparietals:
(0) – absent
(1) – present
(New Character)

342. Epiparietal, number per side not counting midline ep0 or eps:
(0) – five or more
(1) – three
(2) – only one
(Modified from Holmes et al., 2001:28; Farke et al., 2011:57)

343. Epiparietal, presence on midline margin (ep0 locus):
(0) – absent
(1) – present
(New Character)

344. Epiparietal, shape of P0:
(0) – ellipsoidal or crescentic, with constricted base
(1) – triangular, peaked
(New Character)

345. Epiparietal, shape of general epiparietals:
(0) – ellipsoidal or crescentic, often with constricted base
(1) – triangular, peaked
(New Character)

346. Epiparietal, multiple raised undulations on proximal midline ramus of parietal:
(0) – absent
(1) – present
(New Character)

347. Parietal, raised ridge or bumps on dorsal surface of posterior margin (dorsoparietal ridge dpr or dorsoparietal processes dpp):
(0) – absent
(1) – present
(New Character)

348. Epiparietal, raised dorsal undulations on distal midline ramus of parietal:
(0) – absent
(1) – present
(New Character)

349. Parietal, raised bump on midline dorsal surface of posterior margin (dorsoparietal processes dpp0):
(0) – absent
(1) – present
(New Character)

350. Parietal, nodes or raised bumps on dorsal surface of posterior margin in between epiparietal loci ep0 and ep1 (dorsoparietal processes dpp1):
(0) – absent
(1) – present
(New Character)

351. Parietal, nodes or raised bumps on dorsal surface of posterior margin in between epiparietal loci ep1 and ep2 (dorsoparietal processes dpp2):
(0) – absent
(1) – present
(New Character)

352. Parietal, nodes or raised bumps on dorsal surface of posterior margin in between epiparietal loci ep2 and ep3 (dorsoparietal processes dpp3):
(0) – absent
(1) – present
(New Character)

353. Parietal, nodes or raised bumps on dorsal surface of posterior margin in between epiparietal loci ep3 and ep4 (dorsoparietal processes dpp4):
(0) – absent
(1) – present
(New Character)

For the following characters only use one set of 24 from the three following options

NEW Epiparietal Homology Hypothesis of Parsimony (24 characters)

ALL CERATOPSIA See text for usage, do not use with characters (378-425)

354. Epiparietal, ep1 presence:
(0) – present
(1) – absent
(New Character) we consider p1 to be lost in *Rubeosaurus, Einioaurus, Achelosaurus* and *Pachyrhinosaurus.*

355. Epiparietal, ep1 length:
(0) – short
(1) – elongate, forms spike
(New Character) We interpret the dorsal spike in *Anchiceratops, Bravoceratops* and *Regaliceratops* as a coalesced P1. *Bravoceratops* also has a P0.

356. Epiparietal, ep1 spike shape:
(0) – greatly flattened dorsoventrally
(1) – round or oval cross-section
(New Character)

357. Epiparietal, ep1 orientation relative to plane of frill in lateral view:
(0) – in the plane of frill
(1) – curled dorsally or anterodorsally
(New Character)

358. Epiparietal, ep1 lateral curvature:
(0) – in plane of frill
(1) – curved laterally or medially
(New Character)

359. Epiparietal, ep1, curved elements:
(0) – curve laterally
(1) – curve medially
(New Character)

360. Epiparietal, ep2, presence:
(0) – absent
(1) – present
(New Character)

361. Epiparietal, ep2 elongation:
(0) – ep2 is similar to other epiparietals
(1) – elongated into either a triangle, hook or spike
(New Character)

362. Epiparietal, elongated ep2:
(0) – elongated triangular process
(1) – elongated hook or spike
(New Character)

363. Epiparietal, elongated ep2:
(0) – hook about 2 times longer than wide
(1) – spike, elongated more than 4 times longer than wide
(New Character)

364. Epiparietal, ep2 elongated epiparietal shape:
(0) – greatly flattened dorsoventrally
(1) – round or oval cross-section
(New Character)

365. Epiparietal, ep2 orientation in lateral view:
(0) – in plane of the frill
(1) – curled dorsally or anterodorsally
(New Character)

366. Epiparietal, ep2 mediolateral curvature:
(0) – straight
(1) – curled or oriented medially or laterally
(New Character)

367. Epiparietal, ep3, presence:
(0) – absent
(1) – present
(New Character)

368. Epiparietal, ep3 length:
(0) – short, small and crescentic
(1) – elongated into either a triangle, hook or spike
(New Character)

369. Epiparietal, elongated ep3:
(0) – elongated triangular process
(1) – elongated more than 3 times longer than wide
(New Character)

370. Epiparietal, elongated ep3:
(0) – flat
(1) – round
(New Character)

371. Epiparietal, ep3 mediolateral curvature:
(0) – straight
(1) – curled or oriented medially or laterally
(New Character)

372. Epiparietal, ep4, presence:
(0) – absent
(1) – present
(New Character)

373. Epiparietal, ep4 length:
(0) – short, small and crescentic
(1) – elongated into either a triangle, hook or spike
(New Character)

374. Epiparietal, elongated ep4:
(0) – elongated triangular process
(1) – elongated more than 3 times longer than wide
(New Character)

375. Epiparietal, ep4 mediolateral curvature:
(0) – straight
(1) – curled or oriented medially or laterally
(New Character)

376. Epiparietal, ep5, presence:
(0) – absent
(1) – present
(New Character)

377. Epiparietal, shape of the most proximal epiparietal (usually ep6):
(0) – no ep6 or ep7
(1) – small and crescentic
(2) – triangular process
(New Character)

See text for usage, do not use with characters (354-377 or 402-425)

NEW Epiparietal Homology Hypothesis of Parsimony (24 characters)

CENTROSAUR SPECIFIC

378. Epiparietal, ep1 presence:
(0) – present
(1) – absent
(New Character) we consider p1 to be lost in *Rubeosaurus, Einioaurus, Achelosaurus* and *Pachyrhinosaurus.*

379. Epiparietal, ep1 length:
(0) – short
(1) – elongate, forms spike
(New Character) We interpret the dorsal spike in *Anchiceratops, Bravoceratops* and *Regaliceratops* as a coalesced P1. *Bravoceratops* also has a P0.

380. Epiparietal, ep1 spike shape:
(0) – greatly flattened dorsoventrally
(1) – round or oval cross-section
(New Character)

381. Epiparietal, ep1 orientation relative to plane of frill in lateral view:
(0) – in the plane of frill
(1) – curled dorsally or anterodorsally
(New Character)

382. Epiparietal, ep1 lateral curvature:
(0) – in plane of frill
(1) – curved laterally or medially
(New Character)

383. Epiparietal, ep1, curved elements:
(0) – curve laterally
(1) – curve medially
(New Character)

384. Epiparietal, ep2, presence:
(0) – absent
(1) – present
(New Character)

385. Epiparietal, ep2 elongation:
(0) – ep2 is similar to other epiparietals
(1) – elongated into either a triangle, hook or spike
(New Character)

386. Epiparietal, elongated ep2:
(0) – elongated triangular process
(1) – elongated hook or spike
(New Character)

387. Epiparietal, elongated ep2:
(0) – hook about 2 times longer than wide
(1) – spike, elongated more than 4 times longer than wide
(New Character)

388. Epiparietal, ep2 elongated epiparietal shape:
(0) – greatly flattened dorsoventrally
(1) – round or oval cross-section
(New Character)

389. Epiparietal, ep2 orientation in lateral view:
(0) – in plane of the frill
(1) – curled dorsally or anterodorsally
(New Character)

390. Epiparietal, ep2 mediolateral curvature:
(0) – straight
(1) – curled or oriented medially or laterally
(New Character)

391. Epiparietal, ep3, presence:
(0) – absent
(1) – present
(New Character)

392. Epiparietal, ep3 length:
(0) – short, small and crescentic
(1) – elongated into either a triangle, hook or spike
(New Character)

393. Epiparietal, elongated ep3:
(0) – elongated triangular process
(1) – elongated more than 3 times longer than wide
(New Character)

394. Epiparietal, elongated ep3:
(0) – flat
(1) – round
(New Character)

395. Epiparietal, ep3 orientation in lateral view:
(0) – in plane of the frill
(1) – curled dorsally or anterodorsally
(New Character)

396. Epiparietal, ep4, presence:
(0) – absent
(1) – present
(New Character)

397. Epiparietal, ep4 length:
(0) – short, small and crescentic
(1) – elongated into either a triangle, hook or spike
(New Character)

398. Epiparietal, elongated ep4:
(0) – elongated triangular process
(1) – elongated more than 3 times longer than wide
(New Character)

399. Epiparietal, ep4 orientation in lateral view:
(0) – in plane of the frill
(1) – curled dorsally or anterodorsally
(New Character)

400. Epiparietal, ep5, presence:
(0) – absent
(1) – present
(New Character)

401. Epiparietal, shape of the most proximal epiparietal (usually ep6):
(0) – no ep6 or ep7
(1) – small and crescentic
(2) – triangular process
(New Character)

See text for usage, do not use with characters (354-401)

NEW Epiparietal Homology Hypothesis of Parsimony (24 characters)

CHASMOSAUR SPECIFIC

402. Epiparietal, ep1 presence:
(0) – present
(1) – absent
(New Character) we consider p1 to be lost in *Rubeosaurus, Einioaurus, Achelosaurus* and *Pachyrhinosaurus.*

403. Epiparietal, ep1 length:
(0) – short
(1) – elongate, forms spike
(New Character) We interpret the dorsal spike in *Anchiceratops, Bravoceratops* and *Regaliceratops* as a coalesced P1. *Bravoceratops* also has a P0.

404. Epiparietal, ep1 spike shape:
(0) – greatly flattened dorsoventrally
(1) – round or oval cross-section
(New Character)

405. Epiparietal, ep1 orientation relative to plane of frill in lateral view:
(0) – in the plane of frill
(1) – curled dorsally or anterodorsally
(New Character)

406. Epiparietal, ep1 lateral curvature:
(0) – in plane of frill
(1) – curved laterally or medially
(New Character)

407. Epiparietal, ep1, curved elements:
(0) – curve laterally
(1) – curve medially
(New Character)

408. Epiparietal, ep2, presence:
(0) – absent
(1) – present
(New Character)

409. Epiparietal, ep2 elongation:
(0) – ep2 is similar to other epiparietals
(1) – elongated into either a triangle, hook or spike
(New Character)

410. Epiparietal, elongated ep2:
(0) – elongated triangular process
(1) – elongated hook or spike
(New Character)

411. Epiparietal, elongated ep2:
(0) – hook about 2 times longer than wide
(1) – spike, elongated more than 4 times longer than wide
(New Character)

412. Epiparietal, ep2 elongated epiparietal shape:
(0) – greatly flattened dorsoventrally
(1) – round or oval cross-section
(New Character)

413. Epiparietal, ep2 orientation in lateral view:
(0) – in plane of the frill
(1) – curled dorsally or anterodorsally
(New Character)

414. **Epiparietal, ep2 mediolateral curvature:**
(0) – straight
(1) – curled or oriented medially or laterally
(New Character)

415. **Epiparietal, ep3, presence:**
(0) – absent
(1) – present
(New Character)

416. **Epiparietal, ep3 length:**
(0) – short, small and crescentic
(1) – elongated into either a triangle, hook or spike
(New Character)

417. **Epiparietal, elongated ep3:**
(0) – elongated triangular process
(1) – elongated more than 3 times longer than wide
(New Character)

418. **Epiparietal, elongated ep3:**
(0) – flat
(1) – round
(New Character)

419. **Epiparietal, ep3 orientation in lateral view:**
(0) – in plane of the frill
(1) – curled dorsally or anterodorsally
(New Character)

420. **Epiparietal, ep4, presence:**
(0) – absent
(1) – present
(New Character)

421. **Epiparietal, ep4 length:**
(0) – short, small and crescentic
(1) – elongated into either a triangle, hook or spike
(New Character)

422. **Epiparietal, elongated ep4:**
(0) – elongated triangular process
(1) – elongated more than 3 times longer than wide
(New Character)

423. **Epiparietal, ep4 orientation in lateral view:**
(0) – in plane of the frill
(1) – curled dorsally or anterodorsally
(New Character)

424. **Epiparietal, ep5, presence:**
(0) – absent
(1) – present
(New Character)

425. **Epiparietal, shape of the most proximal epiparietal (usually ep6):**
(0) – no ep6 or ep7
(1) – small and crescentic
(2) – triangular process
(New Character)

The following are for epiparietal homology, do not use with characters (354-425)

Epiparietal Homology Hypothesis B: Clayton and Loewen (27 characters)

CENTROSAURINE SPECIFIC

426. Parietal, nodes or raised bumps on dorsal surface of posterior margin (dorsoparietal processes):
(0) – absent
(1) – present
(New Character) Note: we interpret lack of a dpp in *Rubeosaurus, Einiosaurus, Achelosaurus* and *Pachyrhinosaurus canadensis* as evidenced by vestigual dpps present in some specimens of *Pachyrhinosaurus lacustai* and its presence in *Pachyrhinosaurus perotorum*.

427. Epiparietal, ep1 length:
(0) – short
(1) – elongate, forms spike
(New Character) We interpret the dorsal spike in *Anchiceratops, Bravoceratops* and *Regaliceratops* as a coalesced P1. *Bravoceratops* also has a P0.

428. Epiparietal, ep1 spike shape:
(0) – greatly flattened dorsoventrally
(1) – round or oval cross-section
(New Character)

429. Epiparietal, ep1 orientation relative to plane of frill in lateral view:
(0) – in the plane of frill
(1) – curled dorsally or anterodorsally
(New Character)

430. Epiparietal, ep1 lateral curvature in dorsal view:
(0) – in plane of frill
(1) – curved laterally or medially
(New Character)

431. Epiparietal, ep1, curved elements:
(0) – curve laterally
(1) – curve medially
(New Character)

432. Epiparietal, ep2, presence:
(0) – absent
(1) – present
(New Character)

433. Epiparietal, ep2 elongation:
(0) – ep2 is similar to other epiparietals
(1) – elongated into either a triangle, hook or spike
(New Character)

434. Epiparietal, elongated ep2:
(0) – elongated triangular process
(1) – elongated hook or spike
(New Character)

435. Epiparietal, elongated ep2:
(0) – hook about 2 times longer than wide
(1) – spike, elongated more than 4 times longer than wide
(New Character)

436. Epiparietal, ep2 elongated epiparietal shape:
(0) – greatly flattened dorsoventrally
(1) – round or oval cross-section
(New Character)

437. Epiparietal, ep2 orientation in lateral view:
(0) – in plane of the frill
(1) – curled dorsally or anterodorsally
(New Character)

438. **Epiparietal, ep2 mediolateral curvature:**
(0) – straight
(1) – curled or oriented medially or laterally
(New Character)
439. **Epiparietal, ep3, presence:**
(0) – absent
(1) – present
(New Character)
440. **Epiparietal, ep3 length:**
(0) – short, small and crescentic
(1) – elongated into either a triangle, hook or spike
(New Character)
441. **Epiparietal, elongated ep3:**
(0) – elongated triangular process
(1) – elongated more than 3 times longer than wide
(New Character)
442. **Epiparietal, elongated ep3:**
(0) – flat
(1) – round
(New Character)
443. **Epiparietal, ep4, presence:**
(0) – absent
(1) – present
(New Character)
444. **Epiparietal, ep4 length:**
(0) – short, small and crescentic
(1) – elongated into either a triangle, hook or spike
(New Character)
445. **Epiparietal, elongated ep4:**
(0) – elongated triangular process
(1) – elongated more than 3 times longer than wide
(New Character)
446. **Epiparietal, ep5, presence:**
(0) – absent
(1) – present
(New Character)
447. **Epiparietal, shape of the most proximal epiparietal (usually ep6):**
(0) – no ep6 or ep7
(1) – small and crescentic
(2) – triangular process
(New Character)

Epiparietal Homology Hypothesis B: Clayton and Loewen (27 characters) CHASMOSAURINE SPECIFIC

448. **Parietal, nodes or raised bumps on dorsal surface of posterior margin (dorsoparietal processes):**
(0) – absent
(1) – present
(New Character) Note: we interpret lack of a dpp in *Rubeosaurus, Einiosaurus, Achelosaurus* and *Pachyrhinosaurus canadensis* as evidenced by vestigual dpps present in some specimens of *Pachyrhinosaurus lacustai* and its presence in *Pachyrhinosaurus perotorum*.
449. **Epiparietal, ep1 length:**
(0) – short
(1) – elongate, forms spike
(New Character) We interpret the dorsal spike in *Anchiceratops, Bravoceratops* and *Regaliceratops* as a coalesced P1. *Bravoceratops* also has a P0.

450. Epiparietal, ep1 spike shape:
(0) – greatly flattened dorsoventrally
(1) – round or oval cross-section
(New Character)

451. Epiparietal, ep1 orientation relative to plane of frill in lateral view:
(0) – in the plane of frill
(1) – curled dorsally or anterodorsally
(New Character)

452. Epiparietal, ep1 lateral curvature in dorsal view:
(0) – in plane of frill
(1) – curved laterally or medially
(New Character)

453. Epiparietal, ep1, curved elements:
(0) – curve laterally
(1) – curve medially
(New Character)

454. Epiparietal, ep2, presence:
(0) – absent
(1) – present
(New Character)

455. Epiparietal, ep2 elongation:
(0) – ep2 is similar to other epiparietals
(1) – elongated into either a triangle, hook or spike
(New Character)

456. Epiparietal, elongated ep2:
(0) – elongated triangular process
(1) – elongated hook or spike
(New Character)

457. Epiparietal, elongated ep2:
(0) – hook about 2 times longer than wide
(1) – spike, elongated more than 4 times longer than wide
(New Character)

458. Epiparietal, ep2 elongated epiparietal shape:
(0) – greatly flattened dorsoventrally
(1) – round or oval cross-section
(New Character)

459. Epiparietal, ep2 orientation in lateral view:
(0) – in plane of the frill
(1) – curled dorsally or anterodorsally
(New Character)

460. Epiparietal, ep2 mediolateral curvature:
(0) – straight
(1) – curled or oriented medially or laterally
(New Character)

461. Epiparietal, ep3, presence:
(0) – absent
(1) – present
(New Character)

462. Epiparietal, ep3 length:
(0) – short, small and crescentic
(1) – elongated into either a triangle, hook or spike
(New Character)

463. Epiparietal, elongated ep3:
(0) – elongated triangular process
(1) – elongated more than 3 times longer than wide
(New Character)

464. Epiparietal, elongated ep3:
(0) – flat
(1) – round
(New Character)

465. Epiparietal, ep4, presence:
(0) – absent
(1) – present
(New Character)

466. Epiparietal, ep4 length:
(0) – short, small and crescentic
(1) – elongated into either a triangle, hook or spike
(New Character)

467. Epiparietal, elongated ep4:
(0) – elongated triangular process
(1) – elongated more than 3 times longer than wide
(New Character)

468. Epiparietal, ep5, presence:
(0) – absent
(1) – present
(New Character)

469. Epiparietal, shape of the most proximal epiparietal (usually ep6):
(0) – no ep6 or ep7
(1) – small and crescentic
(2) – triangular process
(New Character)

Epiparietal Homology Hypothesis B: Clayton and Loewen (27 characters) ALL CERATOPSIDS

470. Parietal, nodes or raised bumps on dorsal surface of posterior margin (dorsoparietal processes):
(0) – absent
(1) – present
(New Character) Note: we interpret lack of a dpp in *Rubeosaurus, Einiosaurus, Achelosaurus* and *Pachyrhinosaurus canadensis* as evidenced by vestigual dpps present in some specimens of *Pachyrhinosaurus lacustai* and its presence in *Pachyrhinosaurus perotorum*.

471. Epiparietal, ep1 length:
(0) – short
(1) – elongate, forms spike
(New Character) We interpret the dorsal spike in *Anchiceratops, Bravoceratops* and *Regaliceratops* as a coalesced P1. *Bravoceratops* also has a P0.

472. Epiparietal, ep1 spike shape:
(0) – greatly flattened dorsoventrally
(1) – round or oval cross-section
(New Character)

473. Epiparietal, ep1 orientation relative to plane of frill in lateral view:
(0) – in the plane of frill
(1) – curled dorsally or anterodorsally
(New Character)

474. Epiparietal, ep1 lateral curvature in dorsal view:
(0) – in plane of frill
(1) – curved laterally or medially
(New Character)

475. Epiparietal, ep1, curved elements:
(0) – curve laterally
(1) – curve medially
(New Character)

476. Epiparietal, ep2, presence:
(0) – absent
(1) – present
(New Character)

477. Epiparietal, ep2 elongation:
(0) – ep2 is similar to other epiparietals
(1) – elongated into either a triangle, hook or spike
(New Character)

478. Epiparietal, elongated ep2:
(0) – elongated triangular process
(1) – elongated hook or spike
(New Character)

479. Epiparietal, elongated ep2:
(0) – hook about 2 times longer than wide
(1) – spike, elongated more than 4 times longer than wide
(New Character)

480. Epiparietal, ep2 elongated epiparietal shape:
(0) – greatly flattened dorsoventrally
(1) – round or oval cross-section
(New Character)

481. Epiparietal, ep2 orientation in lateral view:
(0) – in plane of the frill
(1) – curled dorsally or anterodorsally
(New Character)

482. Epiparietal, ep2 mediolateral curvature:
(0) – straight
(1) – curled or oriented medially or laterally
(New Character)

483. Epiparietal, ep3, presence:
(0) – absent
(1) – present
(New Character)

484. Epiparietal, ep3 length:
(0) – short, small and crescentic
(1) – elongated into either a triangle, hook or spike
(New Character)

485. Epiparietal, elongated ep3:
(0) – elongated triangular process
(1) – elongated more than 3 times longer than wide
(New Character)

486. Epiparietal, elongated ep3:
(0) – flat
(1) – round
(New Character)

487. Epiparietal, ep4, presence:
(0) – absent
(1) – present
(New Character)

488. Epiparietal, ep4 length:
(0) – short, small and crescentic
(1) – elongated into either a triangle, hook or spike
(New Character)

489. Epiparietal, elongated ep4:
(0) – elongated triangular process
(1) – elongated more than 3 times longer than wide
(New Character)

490. Epiparietal, ep5, presence:
(0) – absent
(1) – present
(New Character)

491. Epiparietal, shape of the most proximal epiparietal (usually ep6):
(0) – no ep6 or ep7
(1) – small and crescentic
(2) – triangular process
(New Character)

Epiparietal Hypothesis C Morphology (9 characters) CENTROSAURINE SPECIFIC

492. Epiparietals, orientation:
(0) – lie in plane of the overall parietal
(1) – at least one epiparietal curls dorsally or anterodorsally out of the plane of parietal
(New Character)

493. Epiparietals, orientation of non-planar elements:
(0) – dorsally oriented
(1) – anteriorly oriented, subparallel to parietal surface
(New Character)

494. Epiparietal, number of non-planar elements per side:
(0) – none
(1) – one
(2) – two or more
(New Character)

495. Epiparietal, orientation of planar elements:
(0) – all are straight, extend perpendicular to parietal margin
(1) – some curve medially or laterally
(New Character)

496. Epiparietal, curvature of planar elements:
(0) – curve in one direction (either medially or laterally)
(1) – curve in multiple
(New Character)

497. Epiparietal, length:
(0) – short
(1) – at least one elongated
(New Character)

498. Epiparietal, hypertrophied elements:
(0) – only one hypertrophied element per side
(1) – two
(2) – three or more
(New Character)

499. Epiparietal, hypertrophied elements, shape:
(0) – morphologically similar to other epiparietals
(1) – transformed into a tapering spike
(New Character)

500. Epiparietal, shape of spike:
(0) – dorsoventrally flattened
(1) – round to oval in cross section
(New Character)

Epiparietal Hypothesis C Morphology (9 characters) CHASMOSAURINE SPECIFIC

501. Epiparietals, orientation:
(0) – lie in plane of the overall parietal
(1) – at least one epiparietal curls dorsally or anterodorsally out of the plane of parietal
(New Character)

502. Epiparietals, orientation of non-planar elements:
(0) – dorsally oriented
(1) – anteriorly oriented, subparallel to parietal surface
(New Character)

503. Epiparietal, number of non-planar elements per side:
(0) – none
(1) – one
(2) – two or more
(New Character)

504. Epiparietal, orientation of planar elements:
(0) – all are straight, extend perpendicular to parietal margin
(1) – some curve medially or laterally
(New Character)

505. Epiparietal, curvature of planar elements:
(0) – curve in one direction (either medially or laterally)
(1) – curve in multiple
(New Character)

506. Epiparietal, length:
(0) – short
(1) – at least one elongated
(New Character)

507. Epiparietal, hypertrophied elements:
(0) – only one hypertrophied element per side
(1) – two
(2) – three or more
(New Character)

508. Epiparietal, hypertrophied elements, shape:
(0) – morphologically similar to other epiparietals
(1) – transformed into a tapering spike
(New Character)

509. Epiparietal, shape of spike:
(0) – dorsoventrally flattened
(1) – round to oval in cross section
(New Character)

Epiparietal Hypothesis C Morphology (9 characters)

510. Epiparietals, orientation:
(0) – lie in plane of the overall parietal
(1) – at least one epiparietal curls dorsally or anterodorsally out of the plane of parietal
(New Character)

511. Epiparietals, orientation of non-planar elements:
(0) – dorsally oriented
(1) – anteriorly oriented, subparallel to parietal surface
(New Character)

512. Epiparietal, number of non-planar elements per side:
(0) – none
(1) – one
(2) – two or more
(New Character)

513. Epiparietal, orientation of planar elements:
(0) – all are straight, extend perpendicular to parietal margin
(1) – some curve medially or laterally
(New Character)

514. Epiparietal, curvature of planar elements:
(0) – curve in one direction (either medially or laterally)
(1) – curve in multiple
(New Character)

515. Epiparietal, length:
(0) – short
(1) – at least one elongated
(New Character)

516. Epiparietal, hypertrophied elements:
(0) – only one hypertrophied element per side
(1) – two
(2) – three or more
(New Character)

517. Epiparietal, hypertrophied elements, shape:
(0) – morphologically similar to other epiparietals
(1) – transformed into a tapering spike
(New Character)

518. Epiparietal, shape of spike:
(0) – dorsoventrally flattened
(1) – round to oval in cross section
(New Character)

Epiparietal Homology Hypothesis D Positional (18 characters) CENTROSAURINE SPECIFIC

519. Epiparietal, ep1, length:
(0) – short, small and crescentic
(1) – elongate, forms spike
(New Character)

520. Epiparietal, ep1 spike shape:
(0) – greatly flattened dorsoventrally
(1) – round or oval cross-section
(New Character)

521. Epiparietal, ep1 orientation relative to plane of frill in lateral view:
(0) – in the plane of frill
(1) – curled dorsally or anterodorsally
(New Character)

522. Epiparietal, ep1 lateral orientation in dorsal view:
(0) – straight
(1) – oriented or curved laterally or medially
(New Character)

523. Epiparietal, ep1 curved elements:
(0) – curve laterally
(1) – curve medially
(New Character)

524. Epiparietal, ep2 presence:
(0) – present
(1) – absent
(New Character)

525. Epiparietal, ep2 elongation:
(0) – short, small and crescentic
(1) – elongated into either a triangle, hook or spike
(New Character)

526. Epiparietal, elongated ep2:
(0) – short process or hook
(1) – elongated into a spike
(New Character)

527. Epiparietal, ep2 shape of elongation:
(0) – flattened dorsoventrally
(1) – round or oval cross-section
(New Character)

528. Epiparietal, ep2 orientation:
(0) – in plane of the frill
(1) – curled dorsally or anterodorsally
(New Character)

529. Epiparietal, ep2 mediolateral curvature:
(0) – in plane of frill
(1) – oriented or curved laterally or medially
(New Character)

530. Epiparietal, ep2, curved elements:
(0) – curve laterally
(1) – curve medially
(New Character)

531. Epiparietal, ep3 presence:
(0) – present
(1) – absent
(New Character)

532. Epiparietal, ep3 length:
(0) – short, shall and crescentic
(1) – elongated into either a triangle, hook or spike
(New Character)

533. Epiparietal, elongated ep3:
(0) – short process
(1) – elongated into a spike
(New Character)

534. Epiparietal, ep3, shape of elongation:
(0) – greatly flattened dorsoventrally
(1) – round or oval in cross-section
(New Character)

535. Epiparietal, ep3 orientation:
(0) – in the plane of the frill
(1) – curled dorsally or anterodorsally
(New Character)

536. Epiparietal, ep4 presence:
(0) – absent
(1) – present
(New Character)

537. Epiparietal, ep4 length:
(0) – short, shall and crescentic
(1) – elongated into either a triangle, hook or spike
(New Character)

538. Epiparietal, elongated ep4:
(0) – short process
(1) – elongated into a spike
(New Character)

539. Epiparietal, ep4 shape elongation:
(0) – greatly flattened dorsoventrally
(1) – round or oval cross-section
(New Character)

540. Epiparietal, shape of the most proximal epiparietal (usually ep7 or ep8):
(0) – only 1 epiparietal, so these positions are absent
(1) – small and crescentic
(2) – developed into triangular flattened spike
(New Character)

Epiparietal Homology Hypothesis D Positional (18 characters) CHASMOSAURINE SPECIFIC

541. Epiparietal, ep1, length:
(0) – short, small and crescentic
(1) – elongate, forms spike
(New Character)

542. Epiparietal, ep1 spike shape:
(0) – greatly flattened dorsoventrally
(1) – round or oval cross-section
(New Character)

543. Epiparietal, ep1 orientation relative to plane of frill in lateral view:
(0) – in the plane of frill
(1) – curled dorsally or anterodorsally
(New Character)

544. Epiparietal, ep1 lateral orientation in dorsal view:
(0) – straight
(1) – oriented or curved laterally or medially
(New Character)

545. Epiparietal, ep1 curved elements:
(0) – curve laterally
(1) – curve medially
(New Character)

546. Epiparietal, ep2 presence:
(0) – present
(1) – absent
(New Character)

547. Epiparietal, ep2 elongation:
(0) – short, small and crescentic
(1) – elongated into either a triangle, hook or spike
(New Character)

548. Epiparietal, elongated ep2:
(0) – short process or hook
(1) – elongated into a spike
(New Character)

549. Epiparietal, ep2 shape of elongation:
(0) – flattened dorsoventrally
(1) – round or oval cross-section
(New Character)

550. Epiparietal, ep2 orientation:
(0) – in plane of the frill
(1) – curled dorsally or anterodorsally
(New Character)

551. Epiparietal, ep2 mediolateral curvature:
(0) – in plane of frill
(1) – oriented or curved laterally or medially
(New Character)

552. Epiparietal, ep2, curved elements:
(0) – curve laterally
(1) – curve medially
(New Character)

553. Epiparietal, ep3 presence:
(0) – present
(1) – absent
(New Character)

554. Epiparietal, ep3 length:
(0) – short, shall and crescentic
(1) – elongated into either a triangle, hook or spike
(New Character)

555. Epiparietal, elongated ep3:
(0) – short process
(1) – elongated into a spike
(New Character)

556. Epiparietal, ep3, shape of elongation:
(0) – greatly flattened dorsoventrally
(1) – round or oval in cross-section
(New Character)

557. Epiparietal, ep3 orientation:
(0) – in the plane of the frill
(1) – curled dorsally or anterodorsally
(New Character)

558. Epiparietal, ep4 presence:
(0) – absent
(1) – present
(New Character)

559. Epiparietal, ep4 length:
(0) – short, shall and crescentic
(1) – elongated into either a triangle, hook or spike
(New Character)

560. Epiparietal, elongated ep4:
(0) – short process
(1) – elongated into a spike
(New Character)

561. Epiparietal, ep4 shape elongation:
(0) – greatly flattened dorsoventrally
(1) – round or oval cross-section
(New Character)

562. Epiparietal, shape of the most proximal epiparietal (usually ep7 or ep8):
(0) – only 1 epiparietal, so these positions are absent
(1) – small and crescentic
(2) – developed into triangular flattened spike
(New Character)

Epiparietal Homology Hypothesis D Positional (18 characters)

563. Epiparietal, ep1, length:
(0) – short, small
(1) – elongate, forms spike
(New Character)

564. Epiparietal, ep1 spike shape:
(0) – greatly flattened dorsoventrally
(1) – round or oval cross-section
(New Character)

565. Epiparietal, ep1 orientation relative to plane of frill in lateral view:
(0) – in the plane of frill
(1) – curled dorsally or anterodorsally
(New Character)

566. Epiparietal, ep1 lateral orientation in dorsal view:
(0) – straight
(1) – oriented or curved laterally or medially
(New Character)

567. Epiparietal, ep1 curved elements:
(0) – curve laterally
(1) – curve medially
(New Character)

568. Epiparietal, ep2 presence:
(0) – present
(1) – absent
(New Character)

569. Epiparietal, ep2 elongation:
(0) – short, small and crescentic
(1) – elongated into either a triangle, hook or spike
(New Character)

570. Epiparietal, elongated ep2:
(0) – short process or hook
(1) – elongated into a spike
(New Character)

571. Epiparietal, ep2 shape of elongation:
(0) – flattened dorsoventrally
(1) – round or oval cross-section
(New Character)

572. Epiparietal, ep2 orientation:
(0) – in plane of the frill
(1) – curled dorsally or anterodorsally
(New Character)

573. Epiparietal, ep2 mediolateral curvature:
(0) – in plane of frill
(1) – oriented or curved laterally or medially
(New Character)

574. Epiparietal, ep2, curved elements:
(0) – curve laterally
(1) – curve medially
(New Character)

575. Epiparietal, ep3 presence:
(0) – present
(1) – absent
(New Character)

576. Epiparietal, ep3 length:
(0) – short, shall and crescentic
(1) – elongated into either a triangle, hook or spike
(New Character)

577. Epiparietal, elongated ep3:
(0) – short process
(1) – elongated into a spike
(New Character)

578. Epiparietal, ep3, shape of elongation:
(0) – greatly flattened dorsoventrally
(1) – round or oval in cross-section
(New Character)

579. **Epiparietal, ep3 orientation:**
(0) – in the plane of the frill
(1) – curled dorsally or anterodorsally
(New Character)

580. **Epiparietal, ep4 presence:**
(0) – absent
(1) – present
(New Character)

581. **Epiparietal, ep4 length:**
(0) – short, shall and crescentic
(1) – elongated into either a triangle, hook or spike
(New Character)

582. **Epiparietal, elongated ep4:**
(0) – short process
(1) – elongated into a spike
(New Character)

583. **Epiparietal, ep4 shape elongation:**
(0) – greatly flattened dorsoventrally
(1) – round or oval cross-section
(New Character)

584. **Epiparietal, shape of the most proximal epiparietal (usually ep7 or ep8):**
(0) – only 1 epiparietal, so these positions are absent
(1) – small and crescentic
(2) – developed into triangular flattened spike
(New Character)

Epiparietal Homology Hypothesis E Sampson (14 characters) CENTROSAURINE SPECIFIC

585. **Epiparietal, ep1, presence:**
(0) – present
(1) – absent, or vestigial
(New Character) Note: we interpret ep1 as missing in *Diabloceratops*, *Albertaceratops*, and *Medusaceratops* and also in *Rubeosaurus, Einiosaurus, Achelosaurus* and *Pachyrhinosaurus canadensis* as evidenced by vestigual ep1s present in some specimens of *Pachyrhinosaurus lacustai* and its presence in *Pachyrhinosaurus perotorum*.

586. **Epiparietal, ep1 length:**
(0) – short, retains general shape of unmodified epiparietals
(1) – elongate, forms spike
(New Character)

587. **Epiparietal, ep1 orientation relative to plane of frill:**
(0) – in the plane of the frill
(1) – curled dorsally or anterodorsally
(New Character)

588. **Epiparietal, ep1 lateral curvature:**
(0) – in the plane of the frill
(1) – curled medially or laterally
(New Character)

589. **Epiparietal, ep2 presence:**
(0) – present
(1) – absent
(New Character)

590. **Epiparietal, ep2 elongation:**
(0) – short, similar to other majority of epiparietals
(1) – elongated into either a hook or spike
(New Character)

591. Epiparietal, ep2 shape of spike:
(0) – dorsoventrally flattened
(1) – round to oval in cross-section
(New Character)

592. Epiparietal, ep2 orientation relative to plane of frill:
(0) – in plane of frill
(1) – curled dorsally or anterodorsally
(New Character)

593. Epiparietal, ep2 mediolateral curvature:
(0) – straight
(1) – curved medially or laterally
(New Character)

594. Epiparietal, ep3 length:
(0) – short, retains general shape of unmodified epiparietals
(1) – elongated
(New Character)

595. Epiparietal, ep3 lateral curvature:
(0) – straight
(1) – curved medially or laterally
(New Character)

596. Epiparietal, ep4, presence:
(0) – absent
(1) – present
(New Character)

597. Epiparietal, ep4 shape:
(0) – short, shall and crescentic
(1) – elongated into either a triangle, hook or spike
(New Character)

598. Epiparietal, elongated ep4 curvature:
(0) – absent
(1) – present
(New Character)

599. Epiparietal, shape of spike:
(0) – flat
(1) – circular in cross-section
(New Character)

600. Epiparietal, ep5 presence:
(0) – absent
(1) – present
(New Character)

601. Epiparietal, ep5 shape:
(0) – short, retains general shape of unmodified epiparietals
(1) – developed into spike
(New Character)

602. Epiparietal, shape of the most proximal epiparietal (usually ep6):
(0) – absent
(1) – small and crescentic
(2) – developed into triangular flattened spike
(New Character)

Epiparietal Homology Hypothesis E Sampson (14 characters) CHASMOSAURINE SPECIFIC

603. Epiparietal, ep1, presence:
(0) – present
(1) – absent, or vestigial
(New Character) Note: we interpret ep1 as missing in *Diabloceratops*, *Albertaceratops*, and *Medusaceratops* and also in *Rubeosaurus, Einiosaurus, Achelosaurus* and *Pachyrhinosaurus canadensis* as evidenced by vestigual ep1s present in some specimens of *Pachyrhinosaurus lacustai* and its presence in *Pachyrhinosaurus perotorum*.

604. Epiparietal, ep1 length:
(0) – short, retains general shape of unmodified epiparietals
(1) – elongate, forms spike
(New Character)

605. Epiparietal, ep1 orientation relative to plane of frill:
(0) – in the plane of the frill
(1) – curled dorsally or anterodorsally
(New Character)

606. Epiparietal, ep1 lateral curvature:
(0) – in the plane of the frill
(1) – curled medially or laterally
(New Character)

607. Epiparietal, ep2 presence:
(0) – present
(1) – absent
(New Character)

608. Epiparietal, ep2 elongation:
(0) – short, similar to other majority of epiparietals
(1) – elongated into either a hook or spike
(New Character)

609. Epiparietal, ep2 shape of spike:
(0) – dorsoventrally flattened
(1) – round to oval in cross-section
(New Character)

610. Epiparietal, ep2 orientation relative to plane of frill:
(0) – in plane of frill
(1) – curled dorsally or anterodorsally
(New Character)

611. Epiparietal, ep2 mediolateral curvature:
(0) – straight
(1) – curved medially or laterally
(New Character)

612. Epiparietal, ep3 length:
(0) – short, retains general shape of unmodified epiparietals
(1) – elongated
(New Character)

613. Epiparietal, ep3 curvature:
(0) – straight
(1) – curved medially or laterally
(New Character)

614. Epiparietal, ep4, presence:
(0) – absent
(1) – present
(New Character)

615. Epiparietal, ep4 shape:
(0) – short, shall and crescentic
(1) – elongated into either a triangle, hook or spike
(New Character)

616. Epiparietal, elongated ep4 lateral curvature:
(0) – absent
(1) – present
(New Character)

617. Epiparietal, shape of spike:
(0) – flat
(1) – circular in cross-section
(New Character)

618. Epiparietal, ep5 presence:
(0) – absent
(1) – present
(New Character)

619. Epiparietal, ep5 shape:
(0) – short, retains general shape of unmodified epiparietals
(1) – developed into spike
(New Character)

620. Epiparietal, shape of the most proximal epiparietal (usually ep6):
(0) – absent
(1) – small and crescentic
(2) – developed into triangular flattened spike
(New Character)

Epiparietal Homology Hypothesis E Sampson *(14 characters)*

621. Epiparietal, ep1, presence:
(0) – present
(1) – absent, or vestigial
(New Character) Note: we interpret ep1 as missing in *Diabloceratops*, *Albertaceratops*, and *Medusaceratops* and also in *Rubeosaurus, Einiosaurus, Achelosaurus* and *Pachyrhinosaurus canadensis* as evidenced by vestigual ep1s present in some specimens of *Pachyrhinosaurus lacustai* and its presence in *Pachyrhinosaurus perotorum*.

622. Epiparietal, ep1 length:
(0) – short, retains general shape of unmodified epiparietals
(1) – elongate, forms spike
(New Character)

623. Epiparietal, ep1 orientation relative to plane of frill:
(0) – in the plane of the frill
(1) – curled dorsally or anterodorsally
(New Character)

624. Epiparietal, ep1 lateral curvature:
(0) – in the plane of the frill
(1) – curled medially or laterally
(New Character)

625. Epiparietal, ep2 presence:
(0) – present
(1) – absent
(New Character)

626. Epiparietal, ep2 elongation:
(0) – short, similar to majority of other epiparietals
(1) – elongated into either a hook or spike
(New Character)

627. Epiparietal, ep2 shape of spike:
(0) – dorsoventrally flattened
(1) – round to oval in cross-section
(New Character)

628. Epiparietal, ep2 orientation relative to plane of frill:
(0) – in plane of frill
(1) – curled dorsally or anterodorsally
(New Character)

629. Epiparietal, ep2 mediolateral curvature:
(0) – straight
(1) – curved medially or laterally
(New Character)

630. Epiparietal, ep3 length:
(0) – short, retains general shape of unmodified epiparietals
(1) – elongated
(New Character)

631. Epiparietal, ep3 lateral curvature:
(0) – straight
(1) – curved medially or laterally
(New Character)

632. Epiparietal, ep4, presence:
(0) – absent
(1) – present
(New Character)

633. Epiparietal, ep4 shape:
(0) – short, shall and crescentic
(1) – elongated into either a triangle, hook or spike
(New Character)

634. Epiparietal, elongated ep4 curvature:
(0) – absent
(1) – present
(New Character)

635. Epiparietal, shape of spike:
(0) – flat
(1) – circular in cross-section
(New Character)

636. Epiparietal, ep5 presence:
(0) – absent
(1) – present
(New Character)

637. Epiparietal, ep5 shape:
(0) – short, retains general shape of unmodified epiparietals
(1) – developed into spike
(New Character)

638. Epiparietal, shape of the most proximal epiparietal (usually ep6):
(0) – absent
(1) – small and crescentic
(2) – developed into triangular flattened spike
(New Character)

Data Placeholders

632. Skull, preorbital skull length compared to overall skull length:

(0) – short, small and crescentic
(1) – elongate, forms spike
(New Character)

633. Episquamosals, number:

(0) – none
(1) – one
(2) – two
(3) – three
(4) – four
(5) – five
(6) – six
(7) – seven
(8) – eight
(9) – nine
(10) – ten
(New Character)

634. Number of alveoli in premaxilla:
635. Number of alveoli in maxilla:
636. Number of alveoli in dentary:
